# Solving the resource constrained project scheduling problem with quantum annealing

**DOI:** 10.1038/s41598-024-67168-6

**Published:** 2024-07-22

**Authors:** Luis Fernando Pérez Armas, Stefan Creemers, Samuel Deleplanque

**Affiliations:** 1 Operations Management, IESEG School of Management, CNRS, UMR 9221 - LEM - Lille Economie Management, Univ. Lille, 3 rue de la digue, 59800 Lille, Nord France; 2 CNRS, Centrale Lille, Junia, Univ. Polytechnique Hauts-de-France, UMR 8520 - IEMN, Univ. Lille, 41 Bd Vauban, 59000 Lille, France; 3 ORSTAT KU Leuven, Naamsestraat 69, 3000 Leuven, Belgium; 4 Center for Operations Research and Econometrics (CORE), Université catholique de Louvain, Voie du Roman Pays 34, 1348 Louvain-la-Neuve, Belgium

**Keywords:** Resource constrained project scheduling problem, Quantum optimization, Quantum annealing, Applied mathematics, Computational science, Computer science, Quantum information

## Abstract

Quantum annealing emerges as a promising approach for tackling complex scheduling problems such as the resource-constrained project scheduling problem (RCPSP). This study represents the first application of quantum annealing to solve the RCPSP, analyzing 12 well-known mixed integer linear programming (MILP) formulations and converting the most qubit-efficient one into a quadratic unconstrained binary optimization (QUBO) model. We then solve this model using the D-wave advantage 6.3 quantum annealer, comparing its performance against classical computer solvers. Our results indicate significant potential, particularly for small to medium-sized instances. Further, we introduce time-to-target and Atos Q-score metrics to evaluate the effectiveness of quantum annealing and reverse quantum annealing. The paper also explores advanced quantum optimization techniques, such as customized anneal schedules, enhancing our understanding and application of quantum computing in operations research.

## Introduction

In the realm of computational methodologies, quantum computing has emerged as a groundbreaking approach, promising revolutionary solutions to intricate optimization problems. At the forefront of this quantum revolution is the paradigm of quantum computing, a computational architecture harnessing the principles of quantum mechanics. At its core lies the quantum bit, or qubit, which exhibits unique properties such as superposition and entanglement, that pave the way for unprecedented computational capabilities. Currently residing in the Noisy Intermediate-Scale Quantum (NISQ)^1^ era, quantum computing is marked by limited-size and noise-sensitive quantum machines. This era acknowledges the ongoing challenges in quantum hardware while emphasizing the potential for valuable research and exploration of quantum advantages, even within the constraints of current technology.

In the landscape of optimization, two prominent approaches dominate the quantum-computing arena. Universal quantum computers that operate using the circuit gate model and showcase proven advantages in algorithms such as Shor’s^2^ and Grover’s^3^, but with limited size (as of 2023, the biggest universal quantum computer has 433 physical qubits but only 414 are available^4–6^). On the other hand, adiabatic quantum computers, grounded in the principle of adiabatic quantum computation, exhibit promising experimental results, particularly in terms of optimization problems by leveraging the ability to map them as energy minimization problems that allow the explotation of the natural tendency of the universe to seek states of minimum energy.

Diverging from gate-based quantum computers, quantum annealers carve a niche with a more focused application scope. These systems boast a higher qubit count (+5000) and enhanced noise resilience, fueling extensive research from both industry and academia. The versatility of quantum annealers is illustrated in their application to complex problems like graph partitioning^7,8^, transportation^9,10^, Job-Shop Scheduling Problem (JSSP)^11,12^, etc^13–16^.

This paper delves into the realm of adiabatic quantum algorithms, with a specific focus on quantum annealing, to tackle one of the most challenging NP-Hard scheduling problems: the Resource Constrained Project Scheduling Problem (RCPSP). The significance of this choice arises from the inherent complexity of the problem, its broad applicability in projects’ makespan minimization, and the non-trivial nature of its formulation as input of the quantum machine. Notably, this work represents the first exploration of applying quantum annealing techniques to the RCPSP.

**Figure 1:**
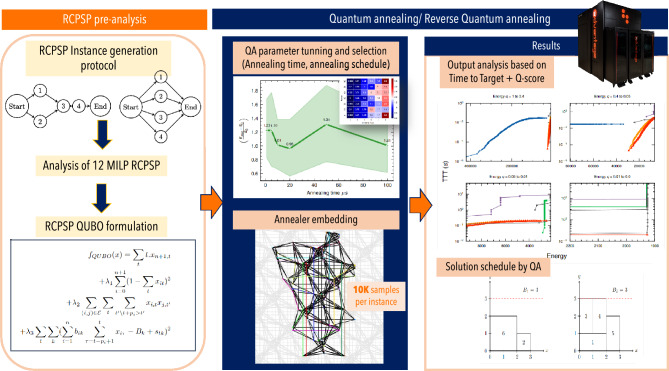
Schematic overview of the methodology employed in this study. The study starts, by searching the most qubit efficient formulation for QA, then moves towards the search of adequate parameters and embedding, to then finally proceed to solve different instances of the RCPSP via QA.

The primary objective of this research is to present a comprehensive analysis of the potential applications and limitations of the current quantum annealing technology. Our study meticulously outlines a step-by-step approach to solving the RCPSP on the cutting-edge D-Wave Advantage quantum annealer featuring 5640 qubits. Through this exploration, we aim to contribute not only to the theoretical understanding of quantum annealing but also to the practical application of this technology in addressing real-world optimization challenges. Figure 1 provides a comprehensive visual representation of the methodology and key processes employed in this study.

To the best of our knowledge, this is the first study to apply quantum annealing to the RCPSP, marking a significant advancement in the application of quantum computing to operations research. The three main contributions of this paper are:

- Evaluation of Mixed Integer Linear Programming (MILP) formulations for the RCPSP: we analyze 12 well-known MILP formulations for the RCPSP, we select the most suitable for QA, and we provide the first corresponding Quadratic Unconstrained Binary Optimization (QUBO) reformulation for this problem.
- Utilization of Time-to-Target (TTT) and Atos Q-score metrics: we introduce these metrics to compare the effectiveness of quantum annealing and reverse quantum annealing against classical optimization techniques. Additionally, we evaluate the largest problem size that can be effectively handled by a modern quantum annealer.
- We adopt advanced quantum optimization techniques: our work discusses advanced methods such as reverse quantum annealing and customized annealing schedules, which are particularly useful for addressing complex combinatorial challenges.

This paper provides an in-depth exploration of quantum annealing’s theoretical and practical aspects, specifically in relation to the RCPSP. Initially, we examine the fundamentals of quantum annealing, focusing on the problem-embedding process into the annealer’s qubit graph. We then explain the RCPSP and detail the methods used in this study, including the selection of instance generators from existing literature, evaluation metrics for quantum heuristics and strategies for utilizing a quantum annealer for RCPSP (annealing time, shots, reverse schedule). The results section presents the QUBO model for solving the RCPSP. Finally, we present an extensive computational experiment to assess the efficacy of quantum annealing when solving the RCPSP and discuss key factors such as solution sampling, annealing duration, chain strength, and the use of advanced controls like reverse annealing. In addition to our conclusions, we also present several promising open research questions.

## Quantum annealing and D-wave machines

Quantum Annealing stands as a quantum metaheuristic designed to tackle combinatorial optimization problems by leveraging the principles of quantum mechanics^17,18^. This approach draws inspiration from simulated annealing^19^, a classical metaheuristic where a system systematically cools to reach a state of minimum energy. In quantum annealing, quantum phenomena such as superposition and quantum tunneling^20^ are harnessed to navigate through local minima efficiently, aiming to pinpoint the global minimum of a cost function. The efficacy of quantum annealing, as compared to its classical counterpart, can vary depending on the specific problem and hardware employed. The literature offers both theoretical proofs^17^, highlighting the advantages of quantum annealing, as well as contrasting perspectives surrounding it in particular simulated annealing^21–23^.

At the core of quantum annealing lies the profound concept of the natural tendency of the universe to seek for states of minimum energy. This method is grounded in the “adiabatic theorem” of quantum mechanics^24,25^ along with the time-dependent Schrödinger equation^26^. It describes the evolution of any quantum system, thereby encapsulating the dynamics of its quantum states. The Hamiltonian, typically represented as a matrix, contains all the pertinent information regarding the quantized energy states available to a quantum system. The adiabatic theorem of quantum mechanics asserts that if a quantum system undergoes a slow and continuous change in its Hamiltonian, it will persist in its instantaneous eigenstate throughout the transformation. In simpler terms, if the system initiates in its ground state and the Hamiltonian changes sufficiently slowly, the system will maintain its ground state.

Applying the adiabatic evolution theorem to optimization problems involves initiating the process with a “simple” initial system represented by the Hamiltonian $\mathcal {H}_0$, for which the ground state can be easily determined. The notations and symbols used throughout this work are reported in Table B1 of Appendix B. The system is then gradually transformed into the problem Hamiltonian $\mathcal {H}_1$, an energetic mapping of an optimization problem $\mathcal {P}$. As the adiabatic evolution concludes, the system originally described by $\mathcal {H}_0$ should have transitioned into the ground state of the problem Hamiltonian, representing the minimum value solution to the optimization problem $\mathcal {P}$. The QA algorithm is physically implemented using analog control devices to manipulate a collection of qubit states following a time-dependent Hamiltonian represented as:

$$\begin{aligned} \mathcal {H}(t) = A(t)\mathcal {H}_0 + B(t) \mathcal {H}_1 \end{aligned}$$

This algorithm orchestrates a gradual transition from an initial ground state in $\mathcal {H}_0$ to a state described by the problem Hamiltonian $\mathcal {H}_1$. The $\mathcal {H}_1$ Hamiltonian mirrors the energy function of the optimization problem, ensuring that the ground state for $\mathcal {H}_1$ corresponds to a minimum-cost solution to the optimization problem $\mathcal {P}$. Introduced by Farhi et al.^27^, QA and in more general the adiabatic quantum model of computation demonstrates that if the transition is executed slowly enough, the algorithm will, with high probability, converge to a ground state, i.e., an optimal solution.

D-Wave quantum annealing processors are purposefully engineered for the specific task of identifying minimum-cost solutions to the Ising Minimization problem or, indirectly, to the QUBO–an isomorphic problem. The Ising problem, defined on a graph $G = (V, E)$, entails the assignment of values from $\{-1, +1\}$ to spin variables $s_i$ with the objective of minimizing the following energy function $\mathcal {H}_1$:

$$\begin{aligned} \mathcal {H}_1 = \sum _i h_i s_i + \sum _{i,j} J_{ij} s_i s_j \end{aligned}$$

Where $h = {h_i: i \in V}$ represent weights, and $J = {J_{ij}: (i, j) \in E}$ is a set of coupling constants. In the physical context, spin variables $s_i$ can be seen as magnetic poles, with negative $J_{ij}$ indicating ferromagnetic interactions, and positive values suggesting antiferromagnetic interactions. The optimal configuration of spin variables $s_i$ that minimizes the energy function is denoted as a ground state, while alternative configurations are classified as excited states that do not have minimum energy. Upon broadening the computational scope, Ising problems are effortlessly transformed into QUBO problems via $s_i = 2x_i - 1$. This modification involves associating binary decision variables $x_i \in \{0, 1\}$ with spin variables $s_i \in \{-1,+1\}$.

A D-Wave Quantum Processor Unit (QPU) maintained at a few millikelvin exhibits quantum properties such as superposition and quantum tunneling. Despite the presence of a Faraday shield, the QPU remains susceptible to interference, which generally reduces the likelihood of attaining a ground state. Consequently, we categorize any D-Wave processor as a heuristic solver, that requires empirical methods for performance analysis. The current most advanced D-Wave processor (Advantage 6.3) has more than 5000 active qubits, a connectivity of 15 qubits (qubits are not fully connected), and 35000 active couplers, made of microscopic loops of niobium. These couplers are connected to a sophisticated analog control system through a network of Josephson junctions^28–30^. Table 1 lists the properties of the different topologies made available by D-Wave: Chimera, Pegasus, and Zephyr.

**Table 1:** Characteristics of available D-wave quantum devices.

Topology	Chimera	Pegasus	Zephyr
Device designation	2000Q	Advantage 6.3	Advantage 2 (Prototype)
Active qubit count	2041	5616	563
Connectivity measure	6	15	20

Besides noise and interference, another major challenge is qubit connectivity. In order to solve any problem on a quantum annealer, the problem graph (QUBO/Ising) must be mapped into the physical hardware, which has a limited number of qubits and interconnections. Consequently, it may be necessary to alter the structure of the QUBO/Ising problem to fit the constrained topology of the quantum annealer^31^. This involves mapping the model onto a larger qubit graph, a critical step that stresses the importance of the machine’s inherent topology. This non-trivial process is known as “minor embedding”.

**Figure 2:**
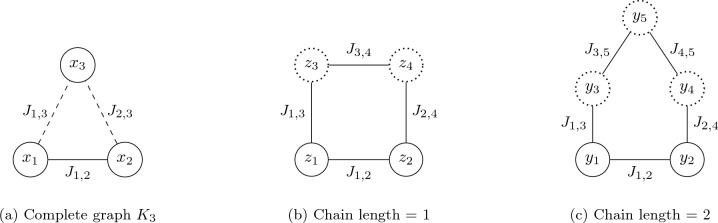
Three graphs representing the same logical QUBO/Ising, but using different embeddings. (**a**) represents the $K_3$ original QUBO/Ising problem. The dashed of the edges (*x*1–$x_3$) and (*x*2–$x_3$) indicate that the corresponding qubits are not physically connected in the QPU. (**b**) shows a minor embedding of (**a**) with the use of two logical qubits *z*3 and *z*4, that can be recognized by their dotted border. (**c**) is a sub-optimal embedding of (**a**) that uses three logical qubits instead of two.

When the QUBO/Ising problem’s graph requires couplings among qubits not directly connected in a QPU topology, additional qubits are utilized to form connected sub-graphs representing the target graph. These additional qubits are referred to as “logical qubits”. For example, consider the QUBO/Ising graph $K_3$ shown in Fig. 2a, where qubits $x_1$ and $x_2$ need to be coupled with $x_3$ but lack direct physical connections in the QPU’s topology, as despicted by the dotted edges between these nodes. Figure 2b illustrates the minor embedding of $K_3$ using a new logical qubit. To establish this logical link, the adjusted QUBO/Ising problem includes an additional penalty term, either $\rho (z_4-z_3)^2$ or $\rho (z_4z_3)$, where $\rho$ denotes the “chain strength parameter” which helps maintain the integrity of logical qubits during computation.

The new logical links established by the minor embedding process are termed “chains”. For the solution to be consistent, it is critical that all physical qubits within a chain attain the same value upon measurement; failure to do so indicates a broken chain. The “chain strength parameter” $\rho$ manages a crucial trade-off: setting it too low risks frequent chain breaks, whereas a value that is set too high may restrict the qubits capacity to transition between states.

Sub-optimal embeddings can lead to unnecessarily long chains, as depicted in Fig. 2c. This embedding requires the use of two additional logical qubits, along with the inclusion of extra penalty terms $\rho (y_4y_3)$ and $\rho (y_4y_5)$, thereby increasing the likelihood of chain breaks. The presence of multiple broken chains often indicates that the solutions yielded by the annealing process may not accurately represent the original problem. As demonstrated by Marshal et al.^32^, poor embeddings, particularly those characterized by long chains, can complicate the solution sampling process. Although various post-processing techniques exist to mend broken chains, such as majority voting or Monte Carlo resampling, this work exclusively considers unbroken chains. Samples with broken chains were discarded, impacting only runtime performance and not the number of valid solutions, regardless of whether discarded samples were feasible or not.

**Figure 3:**
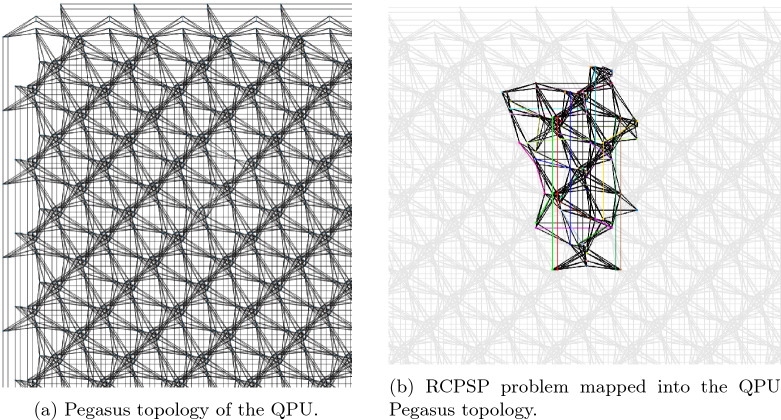
Minor embedding process with the Pegasus topology.

A wide range of heuristics for finding embeddings are available in the literature. Moreover, the quest for better embedding algorithms remains an active area of research^33–35^. Notably, Bernal et al.^36^ have demonstrated that more effective embeddings can be achieved through integer programming and decomposition methods. In this study, minor embeddings were identified using D-Wave’s available heuristic^37^. Figure 3 illustrates an example of the embedding process using the “minor-miner” heuristic on the “Pegasus” topology.

Once the minor embedding process is successfully completed, the annealing process is initiated. While the adiabatic theorem suggests a potentially lengthy annealing duration to ensure the system stays in the ground state, practical constraints arise due to noise, which can elevate the system to (unwanted) higher energy states. Consequently, a brief annealing is conducted and repeated multiple times in a stochastic process that samples from the energy distribution of the problem. Determining the optimal annealing time and the number of samples depends on the specific problem and its energy distribution. Typical values fall within the range of 10 to 100 microseconds for annealing times and 1 to 10000 for the number of samples.

While the traditional QA starts from the ground state of the “Ising Transverse field” Hamiltonian $\mathcal {H}_0$, and therefore from an initial uniform superposition and evolves towards the target problem Hamiltonian $\mathcal {H}_1$, a more sophisticated evolution approach can be applied. In Reverse Annealing (RA), the process departs from an already available solution. RA reintroduces partial segments of $\mathcal {H}_0$ by operating in reverse, thereby reinstating a partial quantum superposition. This unique reverse progression is employed to iteratively refine the initial solution^38–41^.

## Resource-constrained project scheduling problem

The RCPSP is one of the most extensively studied scheduling problems and perhaps one of the easiest to describe; however despite this apparent simplicity, the RCPSP conceals its true complexity, as demonstrated by Blazewicz et al.^42^, who show that the RCPSP is NP-hard. This inherent complexity renders the RCPSP as one of the most intractable combinatorial optimization problems. Similar to the JSSP^43,44^ the RCPSP falls into the category of problems classified as NP-hard “in the strong sense”^45^.

In essence, the RCPSP considers the scheduling of a single project comprising $n$ non-dummy activities, subject to precedence and resource constraints, with the overarching objective of minimizing the project makespan–the total time required for the completion of all activities. The RCPSP is typically represented by a graph $G(\mathcal {A},\mathcal {E})$ with each node in $\mathcal {A} = \{0, 1, ..., n + 1\}$ that corresponds to the different project activities and each edge in $(i, j) \in \mathcal {E}$ equivalent to a straightforward finish-to-start precedence relationship, meaning that the beginning of a successor activity $j$ must await the completion of its predecessor activity $i$. Nodes $0$ and $n+1$ serve as symbolic milestones, representing the “project start” and “project finish”, respectively. These milestone activities are often referred to “dummy activities”. Each activity $j \in \mathcal {A}$ has a duration $p_j$ and resource consumption $b_{jk}$, where *k* belongs to a set of renewable resources $\mathcal {R}$. Each resource *k* has a maximum capacity $B_k$. A feasible solution to the RCPSP corresponds to a project schedule $\mathcal {S} = \{S_0,S_1,\dots ,S_{n+1}\}$ comprised of start times $S_j$ (for each activity $j \in \mathcal {A}$) that respect both precedence and resources constraints. An inherent characteristic of the RCPSP is the non-preemptive nature of activities, indicating that once an activity commences, it cannot be interrupted.

**Figure 4:**
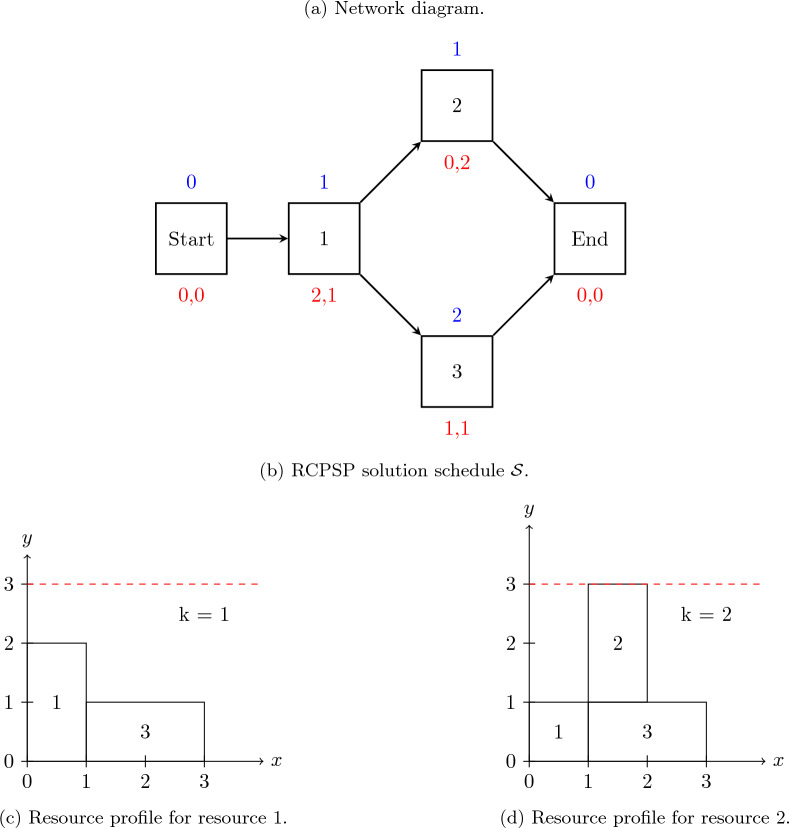
RCPSP instance example: (**a**) RCPSP instance graph $G(\mathcal {A},\mathcal {E})$ with three non-dummy activities and two resources; (**b**) solution schedule for the instance; (**c**) resource profile for resource 1, and (**d**) resource profile for resource 2.

Figure 4a depicts the graphical representation of a small instance of RCPSP (known as a network diagram) composed of three activities (plus two “dummy” activities) and two renewable resources. The activity duration, denoted as $p_j$, and resource consumption $b_{jk}$ are presented above and below each node *j*, respectively. Both renewable resources have a maximum capacity of three units. Figure 4b shows the optimal schedule $\mathcal {S} = \{S_0 = 0, S_1 = 0, S_2 = 1, S_3 = 1, S_4 = 3\}$ for this instance.

### Mixed integer linear programming formulations

Given the intrinsic significance of the RCPSP, it is unsurprising that the literature dedicated to Mixed Integer Linear Programing (MILP) formulations for the RCPSP is both prolific and dynamic, offering a myriad of approaches. For a given optimization problem, multiple formulations can be devised. These can be distinguished in three ways: first by the formulation size, notably concerning the number of variables; secondly, by the number of constraints they involve; and thirdly, by the strength of their Linear Programming (LP) relaxation. Typically, for a given problem, a discernible correlation exists between the problem size and the quality of the LP relaxation. Traditionally, enhancing the quality of the LP relaxation requires a new and extended formulation that introduces additional variables and constraints, thus increasing the problem size. With remarkable advancements in LP algorithms, which can now efficiently solve instances involving millions of variables^46–48^, it comes as no surprise that, in the context of MILP formulations for the RCPSP, a significant emphasis has been placed on advancing and refining the quality of the LP relaxations^49,50^.

**Figure 5:**
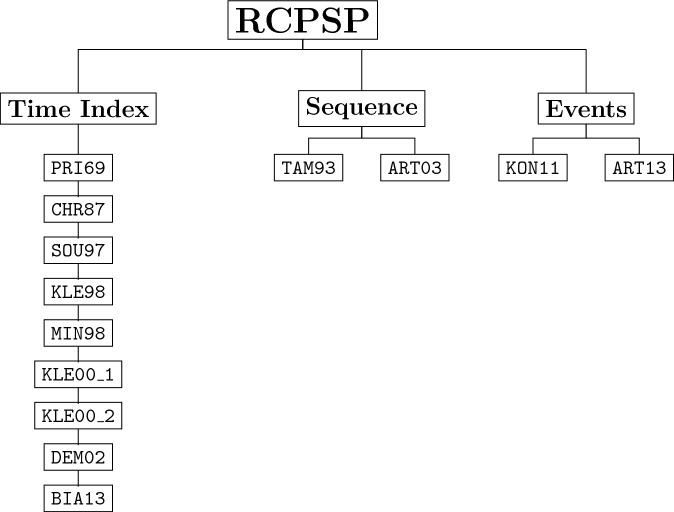
RCPSP MILP formulations reported in the literature categorized by their family type.

Broadly classified, formulations for the RCPSP fall into three distinct families, as illustrated in Fig. 5. The first category encompasses time-index formulations^51–57^, including works by Pritsker et al. 1969 (PRI69), Christofides et al. 1987 (CHR87), de Sousa and Wolsey 1997 (SOU97), Mingozzi et al. 1998 (MIN98), Klein and Kaplan 1998 (KLE98), Klein 2000 (KLE00_1 and KLE00_2), Demeulemeester and Herroelen 2002 (DEM02), and Bianco and Caramia 2013 (BIA13). This is followed by sequence-based formulations^58,59^, exemplified by Tamarit and Valdés 1993 (TAM93) and Artigues et al. 2003 (ART03).The third and final category comprises event-based formulations^60,61^, including works by Koné et al. 2011 (KON11) and Artigues et al. 2013 (ART13). An analysis by Koné and Artigues^61,62^ reveals that sequence-based and event-based formulations tend to be more compact, requiring fewer variables, while time-index formulations, although larger in scale, offer superior Linear Programming (LP) relaxations. It is noteworthy that the majority of time-index formulations tend to be binary and utilize exclusively binary variables, while event-based and sequence-based formulations employ a mix between integer and binary variables. For more general information regarding the different MILP formulations of the RCPSP, please refer to Artigues et al 2013^63^.

In the context of employing a quantum annealer for solving the RCPSP, a pertinent question emerges: Which formulation should one choose among the available options? Specifically, which formulation aligns most effectively with the capabilities of current commercially accessible quantum annealers? Addressing these inquiries necessitates an investigation into the transformation of these formulations into their corresponding QUBO form. This evaluation involves assessing each formulation based on factors such as the resulting QUBO size, the need for additional slack-supplementary variables, and the sparsity of the associated QUBO graph. These characteristics play a pivotal role in determining the suitability of each formulation for solving the RCPSP, particularly within the confines of NISQ-era quantum annealers. This study aims to provide comprehensive insights to answer these critical questions.

## Methods

In the following section, we explain different aspects of the experimental methodology followed in this study.

### RCPSP instance selection protocol

A classical study for assessing the performance of solving the RCPSP would normally involve instances from the well-known PSPLIB dataset of Kolisch et al.^64,65^ or alternatively, the more recent CV dataset of Coelho and Vanhoucke^66^. Both datasets offer a large number of hard instances of varying sizes (where size is defined by the number of project activities, ranging from 20 to 120 activities). However, considering the limitations on the number of available qubits in the D-wave Advantage 6.3 Quantum Annealer, we decided to utilize the RanGen instance generator proposed by Demeulemeester, Vanhoucke, and Herroelen^67,68^. This generator allows the creation of random instances with varying levels of difficulty.

The instances used on this work were generated following the well-established protocol proposed by Baptiste and Le Pape^69^, which involves constructing both disjunctive and cumulative instances. Disjunctive instances are characterized by a large number of precedence constraints, resulting in a highly sequential schedule with limited opportunities for parallel execution. On the other hand, cumulative instances exhibit fewer precedence constraints and provide ample opportunities for parallelism and posing a greater challenge for solving.

**Figure 6:**
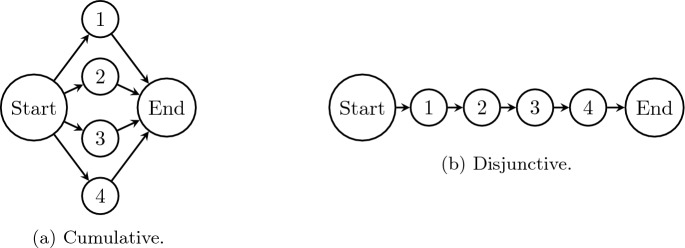
Baptiste and Le Pape instance types: (**a**) the left graph corresponds to a cumulative RCPSP instance; (**b**) right graph corresponds to a disjunctive RCPSP instance.

The RanGen generator facilitates the generation of both disjunctive and cumulative instances using the “Order Strength” (OS) parameter, ranging from 0 to 1. A value of 0 indicates a fully cumulative instance, illustrated in Fig. 6a, while 1 represents a fully disjunctive instance, illustrated in Fig. 6b. The generator also allows tuning the resource constraints by specifying the number of resources, in addition to the level of constraint for renewable resources. This is done via a parameter called “Resource Constrainedness” (RC) that again ranges from 0 to 1, where 1 means that all activities consume the maximum resource capacity $B_k$, while 0 means that there is no resource consumption by the project activities.

In our study, we utilized RanGen to produce instances of the RCPSP across various dimensions determined by the number of non-dummy activities (i.e., $n \in \left\{ 3, 4, 5, 6, 7, 8 \right\}$). Within each size category, we generated three distinct instance types characterized by OS values of 0.1, 0.5, and 0.9. We refer to these instance types as “Cumulative”, “Medium OS”, and “Disjunctive”, respectively. Each instance features two renewable resources (*k* = 2) with a RC set to 0.5. The activity duration $p_j$ and resource consumption values $b_{jk}$ were constrained within the range of 1 to 2 units to further limit the number of qubits required by the quantum annealer. Furthermore, for every instance, the maximum resource capacity $B_k$ was established at 3 units.

### Benchmarking metrics for quantum optimization

Benchmarking metrics play a pivotal role in the evaluation of adiabatic evolution computing algorithms, particularly for quantum annealing. In the context of benchmarking, there are inherent challenges that arise from the unique nature of quantum annealers. First, QA blends quantum and analog elements that are devoid of discrete instructions or basic operations that lend themselves to conventional counting methods used by classical computers. Consequently, given the transient and unstable nature of QA, relying on runtime as an evaluation metric becomes a pragmatic approach. Specially when considering that runtime is widely recognized as one of the most critical metrics for assessing algorithm performance.

A further complication arises from the juxtaposition of hardware-implemented quantum annealing algorithms against their software-implemented counterparts (simulated quantum annealing). Traditional benchmarks for computer platforms, software, and algorithms often fail to account for this mixed scenario, leading to a deficiency in standard guidelines for robust benchmarking.

Unlike classical benchmarks that often distill performance into a single metric, the evaluation of adiabatic evolution algorithms, particularly in the quantum realm, necessitates a more nuanced approach. The performance of a heuristic on a given input is aptly described by a curve delineating the trade-off between computation time and solution quality. This nuanced perspective requires a repertoire of metrics for comprehensive evaluation.

In the empirical evaluation of quantum annealing and adiabatic evolution algorithms, several performance metrics have surfaced in the literature. Notable among these is “Time-to-Solution”^70^ (TTS), which centers on the total time required for a solver to identify a ground state (optimal solution) with a sufficiently high probability. One of the biggest disadvantages of TTS, is that it relies on a priori knowledge of the optimal solution, potentially overlooking benefits derived from near-ground state solutions.

Alternatively, “Time-to-Target” (TTT)^71^ is a relevant metric that focuses on the overall time required by solvers to attain a target solution energy, which is determined by the energy distribution of the quantum annealing processor. This metric provides a more versatile evaluation and acknowledges solutions that may not align with a predetermined optimal outcome.

Another recent interesting metric is the “Q-score”^72^ conceived by the technology consulting company Atos. The Q-score measures the maximum number of qubits effectively employed to solve combinatorial optimization problem. Originally, the Q-score was developed to evaluate the solution of the Max-Cut problem using the Quantum Approximate Optimization Algorithm^73^ (QAOA); however, this metric can be readily adapted to assess the performance of quantum annealing, demonstrated by Van der Schoot et al.^67^. The Q-score of a given problem can be then calculated using:

1 $$\begin{aligned} n^{*} \equiv \max \{n \in \mathbb {N}, \beta (n) > \beta ^* \}. \end{aligned}$$

With $\beta ^* = 0.2$ (obtained empirically by studying the behavior of QAOA). and $\beta (n)$ determined by the following ratio:

2 $$\begin{aligned} \beta (n) = \frac{\tilde{C}(n) - C_{r}(n)}{C_{\max }(n) - C_{r}(n)}. \end{aligned}$$

Where $\tilde{C}(n)$ is the average energy output obtained from QA, $C_{r}(n)$ is the average output value obtained from solving the QUBO/Ising problem with a random sampling method, and $C_{\max }(n)$ equal to the energy of the ground state.

For achieving specific goals of this paper, metrics of interest include TTT and the Q-score. By concentrating on these, we aim to ascertain the potential for speed-ups and determine the largest instance of the RCPSP, measured in terms of the number of project activities, that can be effectively addressed using the current Advantage 6.3 Quantum Annealing system from D-Wave.

### QUBO penalty selection strategy

Adjusting the values of the multipliers by weighing the penalties due to relaxed constraints is an important but difficult step. Given a QUBO problem where $E(x) = x^TQx$, with a number of *n* binary variables $x_i, \forall i \in \mathcal {X}$, we can further divide it into two portions: $E(x) = v(x) + \lambda c(x)$, where *v*(*x*) corresponds to the energy contribution to the objective function, *c*(*x*) corresponds to the energy contribution due to the problem constraints, and $\lambda$ represents the penalty weight. The main goal this becomes finding the value of $\lambda$ such that the optimal solution to the penalised objective function is the optimal solution of the original constrained problem. Multiple strategies have been proposed in the literature, each offering a unique approach to determining the most effective penalty coefficients.

One of the earliest and simplest strategies, proposed by Lucas in 2014^74^, involves utilizing the upper bound of the pure objective function. This is mathematically represented as $\lambda = x^TQx$, where $x_i = 1, \forall i \in \mathcal {X}$ (i.e., the solution where all binary decision values are set to 1 is used as an upper bound of the energy minimization problem). This approach provides a straightforward and easily computable penalty.

Lucas also introduced another possibility for penalty selection which involves using the maximum QUBO value; this is denoted as:

3 $$\begin{aligned} max (Q_{ij}) \quad \forall \quad (i, j) \in \mathcal {X}^2. \end{aligned}$$

This method takes into account the highest interaction value between the variables, ensuring that the penalty is significant enough to enforce the constraints effectively.

More recently, Verma and Lewis^75^ proposed a more sophisticated method in 2020. Their approach estimates the potential gain or loss in the objective function value that could result from switching a particular bit on or off. This method provides a more nuanced and dynamic way of calculating penalties, potentially leading to more accurate and efficient solutions, especially in complex scenarios where the impact of each binary variable on the objective function is not uniform.

4 $$\begin{aligned} W_c= & {} \left\{ \begin{array}{ll} -Q_{ii} - \sum _{\begin{array}{c} j=1 \\ j \ne i \end{array}}^{n} \min \{Q_{ij}, 0\}, Q_{ii} + \sum _{\begin{array}{c} j=1 \\ j \ne i \end{array}}^{n} \max \{Q_{ij}, 0\} \quad \forall i \in \mathcal {X} \end{array} \right\} , \end{aligned}$$

5 $$\begin{aligned} \lambda= & {} \max _{i=1}^{n} W_{c_i}. \end{aligned}$$

Therefore the array of strategies discussed underscores the dynamic and continuously evolving landscape of penalty selection in QUBO problems. Each approach, distinct in its methodology, has its own set of strengths and is tailored to suit different problem types. As penalty selection stands as a vibrant and ongoing field of research, our study opts for a more foundational approach. We employ a simple penalty selection method where the penalties are just a multiple of the sum of activity duration’s $p_i$, $\lambda = \sum _{j=1}^{n} p_i$, leveraging the upper bound strategy, to ensure clarity and ease of implementation during our analysis.

### Experimental setup

The experimental results presented in this study were derived from the utilization of the D-Wave Advantage 6.3 Quantum Annealer, featuring 5640 qubits and a connectivity of 15 connections per qubit. The minor embeddings were established employing D-Wave’s “minor-miner” heuristic. Each instance generated using the approach explained in “Experimental results” section was solved on the QPU with a specific annealing time of $20 \mu s$ (the decision to use $20 \mu s$ is based on the analysis conducted in “Anneal time and pausing effects” section and is also shown in Fig. 18). Furthermore, for every instance, 10000 samples were recorded. It is important to note that the mentioned annealing time exclusively pertains to quantum annealing and does not extend to Reverse Quantum Annealing (RQA), since the latter uses an specific annealing schedule described next.

**Figure 7:**
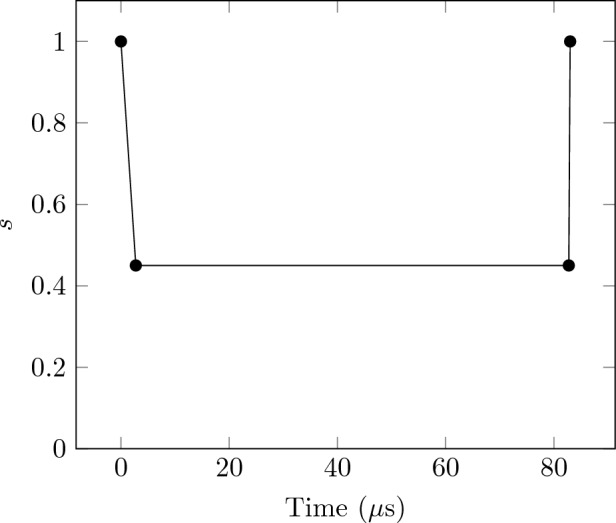
Reverse quantum annealing schedule used for the RQA numerical results of this work.

Figure 7 illustrates the implementation of RQA using a 4-point schedule: [[0.0, 1.0], [2.75, 0.45], [82.75, 0.45], [83.025, 1.0]]. The schedule involves a reverse evolution from $s=1$ to $s=0.45$ within the initial 2.75 ${\upmu }$s, followed by an 80 ${\upmu }$s pause. Subsequently, a forward evolution of 1 ${\upmu }$s occurs, transitioning from $s=0.45$ to $s=1$. Here, the variable *s* represents the percentage of implementation of the problem Hamiltonian $\mathcal {H}_1$ during the annealing evolution.

**Figure 8:**
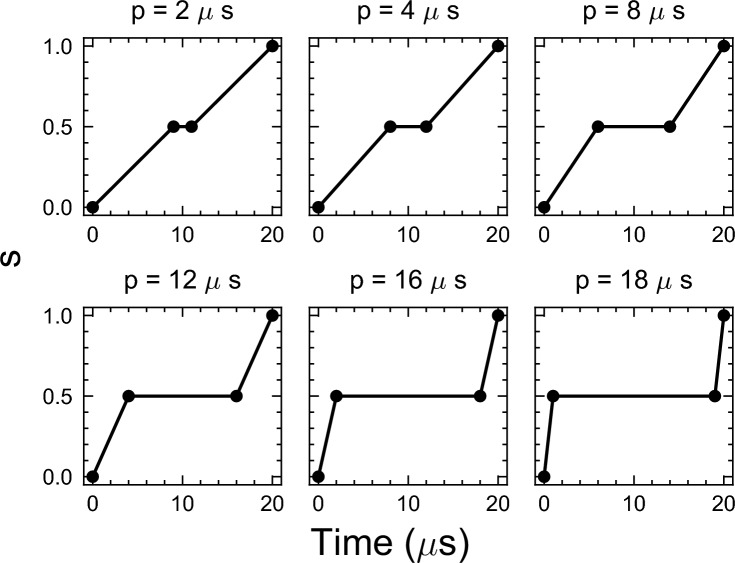
Annealing schedules with pauses of different lengths (2,4,8,12,16, and 18$\mu s$) for a fixed annealing time of 20$\mu s$. These pauses correspond then to a 10%, 20%, 40%, 60%, 80%, and 90% pause of the annealing time.

The impact of including pauses in the annealing schedule on the performance of QA was assessed in “Anneal time and pausing effects” section. An annealing time of 20$\mu$s was employed, incorporating pauses of 2, 4, 6, 8, 12, 16, and 18$\mu$s, respectively. The resulting annealing schedules are visualized in Fig. 8. This analysis was comprehensive, and covered all instance types and sizes. The evaluation involved recording the relative difference between the energy of the ground state $E_0$ and the minimum energy value achieved by QA, denoted as $E_{\min }$. This difference is calculated as $\left( \frac{E_{\min } - E_0}{E_0}\right)$. For each instance, 1000 samples were recorded. A similar analysis was conducted to evaluate the effect of different annealing times 1, 5, 10, 20, 50, and 100$\mu s$ in the performance of QA.

To create a comprehensive benchmark for assessing the performance of QA and RQA in this study, we have selected the following classical optimization techniques: Random Sampling (RS) and Simulated Annealing^19^ (SA), along with classical solvers including GUROBI, COIN-CBC, and GLPK.

## Results

In this section, we provide a detailed examination of several MILP formulations and discuss their applicability in quantum annealing. Initially, in “QUBO analysis of MILP formulations for the RCPSP” section, we explore twelve MILP formulations for the RCPSP to identify the most qubit-efficient model suitable for quantum annealing. This analysis leads us into “RCPSP QUBO” section, where the chosen MILP formulation is transformed into a QUBO form. “Experimental results” section then presents the experimental results obtained from evaluating the performance of QA and RQA across various RCPSP instances, based on the QUBO developed in the preceding section. Finally, “Anneal time and pausing effects” section showcases additional experimental tests that investigate the impact of pausing and annealing times on the effectiveness of the quantum annealing.

### QUBO analysis of MILP formulations for the RCPSP

In this section, we identify the optimal QUBO formulation for the RCPSP, focusing on the formulation that requires the fewest qubits. The minimal number of qubits is directly related to the number of slack variables, the type of variables, and the sparsity of the QUBO graph. Initially, we examine these crucial elements across three types of RCPSP formulations. Subsequently, from the eight formulations studied within the selected type, we determine the most effective one.

#### Analysis of formulation types

**Figure 9:**
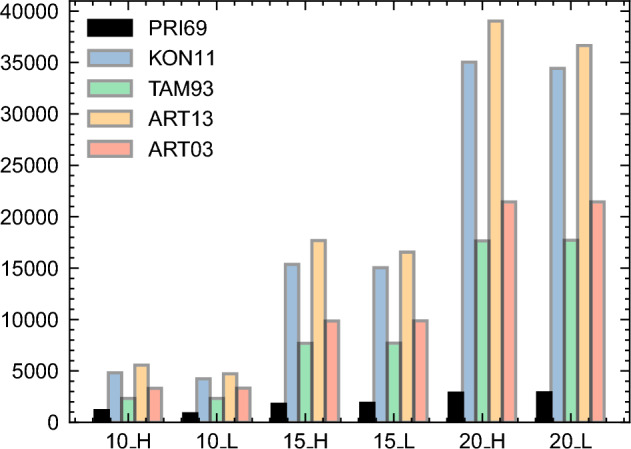
Maximum number of qubits required by the different families of the RCPSP MILP formulations. H refers to disjunctive instances that have high OS (i.e., OS = 0.9), while L refers to cumulative instances that have low OS (i.e., OS = 0.1).

Once transformed into QUBO formulations, time-index, sequence, and event formulations (see Fig. 5) are compared with the data presented in Table C2. The number of qubits required for each formulation is reported in Fig. 9, which shows that the time-index formulation PRI69 requires significantly fewer qubits compared to the sequence- and event-based formulations. Moreover, the gap between these formulations increases with the the instance size. These results may appear counter-intuitive when considering the original number of variables required by each formulation, as detailed in Table C2. Notably, sequence- and event-based formulations exhibit a lower number of original variables than time-index formulations; the compactness characteristic of event-based formulations has been documented well by Koné^62^.

The apparent discrepancy in the number of QUBO variables can be elucidated by closely inspecting the nature of the different formulations. The TAM93 formulation requires *a priori* knowledge of “forbidden sets” $\mathcal {F}$. These are sets of activities that share no precedence constraints and, when scheduled parallely, violate resource constraints. Demeulemeester and Herroelen 2006^76^ demonstrated that generating the minimum forbidden sets entails a worst-case complexity of $O(2^n)$. Consequently, it logically demands more qubits compared to PRI69. This is particularly the case when instance size increases, since the size of the forbidden sets $\mathcal {F}$ increases accordingly.

In the ART03 resource flow formulation, instead of using forbidden sets $\mathcal {F}$, integer variables $\phi ^k_{ij}$ (where $i,j \in \mathcal {A}^2$ and $k \in R$) are employed, each requiring $\lfloor \log _2 \phi ^k_{ij} \rfloor +1$ binary qubits for transformation. These variables take part of multiple inequalities for maintaining resource constraints, leading to the need for additional slack integer variables, which also require binary transformation. Similarly, event-based formulations KON11 and ART13 utilize integer variables $t_e$ to represent event start times, with KON11 also incorporating extra integers $b_{ek}$ for resource consumption. These variables, also used in several constraints, require further slack variables for binary conversion.

#### Analysis of time-index formulations

Taking the same RCPSP instances as before, Table C3 presents the outcome resulting from the conversion of eight distinct RCPSP time-index MILP formulations into QUBO.

**Figure 10:**
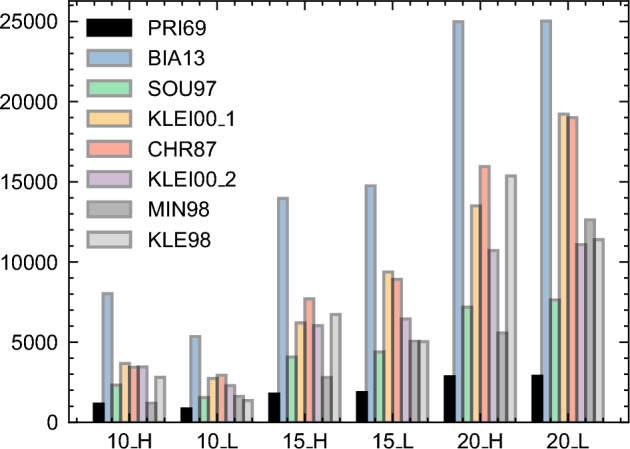
Maximum number of qubits required by different time-index MILP formulations for the RCPSP. H refers to disjunctive instances that have high OS (i.e., OS = 0.9) and L refers to cumulative instances that have low OS (i.e., OS = 0.1).

The number of qubits per formulation is represented by Fig. 10, which shows the efficiency of the PRI69 formulation in comparison to other time-index formulations. This outcome is unsurprising given the inherent simplicity of PRI69 that is characterized by fewer variables, a diminished count of inequalities, and consequently, a reduced number of slack variables. The next favorable option is the MIN98 formulation; however, this choice entails the prerequisite knowledge of feasible sets $\mathcal {I}$. Similar to TAM93, the determination of these sets $\mathcal {I}$ entails a worst-case complexity of $O(2^n)$.

For the other formulations, the various strategies aimed at optimizing solutions for a classical computer prove to be costly in terms of the number of qubits required. We can highlight a few of them here: the CHR87 formulation modifies the PRI69 formulation by replacing its precedence constraint with additional inequalities; SOU97 introduces “step” variables to signal the start of an activity, while KLE98 considers a “step” formulation based on the MILP of a preemptive RCPSP^56^; The KLE00_1 and KLE00_2 formulations incorporate “on/off” variables; Lastly, BIA13 integrates continuous binary variables indicating the percentage of activity completion, while MIN98, bearing similarities to TAM93, requires prior knowledge of “feasible sets” $\mathcal {I}$.

### RCPSP QUBO

Pritsker et al.^51^ proposed PRI69 one of the first formulations for the RCPSP. Although it may initially seem counterintuitive, we have chosen this formulation as the basis for constructing our QUBO based on the analysis in the previous section. We use the notations given in Table 2 to introduce the parameters.

**Table 2:** RCPSP parameters used in the PRI69 time-index formulation.

Notation	Definition
*n*	Number of activities (excluding the two dummy activities indexed by 0 and $n+1$).
$\mathcal {T}$	Maximum number of time periods.
$\mathcal {A}$	Set of activities.
$\mathcal {H}$	$\mathcal {H} = {0, 1,..., T}$ is the scheduling horizon.
$\mathcal {E}$	Set of edges representing a precedence relationship between two activities. For instance, if *i* and *j* are two activities of $\mathcal {A}$, and if activity *i* must be finished to start activity *j*, $(i,j) \in \mathcal {E}$.
$\mathcal {R}$	Set of resources.
$p_i$	Processing time of the activity $i \in \mathcal {A}$.
$B_k$	Capacity of the resource $k, k \in \mathcal {R}$.
$b_{ik}$	Activity *i* consumption of the resource $k \in \mathcal {R}$ and its capacity $B_k$.

A single type of binary decision variable is considered, denoted as $x_{it}$, with $i \in \mathcal {A} \cup \{0, n+1\}$ and $t \in \mathcal {H}$. This variable is indexed by both the activities and the associated time. Each element $x_{it}$, $\forall i \in \mathcal {A} \cup \{0, n+1\}$ and $\forall t \in \mathcal {H}$, takes a $\{0;1\}$ value such that:

$$\begin{aligned} x_{it} = \left\{ \begin{array}{ll} 1 &{} \text{ if } \text{ activity } \text{ i } \text{ starts } \text{ at } \text{ period } t, \\ 0 &{} \text{ otherwise. } \end{array} \right. \end{aligned}$$

The initial objective function of^51^ minimizes the starting time of the dummy-end activity. This function is noted *f*(*x*) and its expression is given by (6). This is the basis of our QUBO, which will then be completed with penalties corresponding to the relaxations of the constraints.

6 $$\begin{aligned} f(x) = \sum _t t x_{(n+1)t}. \end{aligned}$$

Moreover, we also consider the work of^11,12^ on the JSSP, especially for the precedence constraints and the one-start constraint reformulation. For the latter, we force each activity to start exactly once with the following set of constraints (7).

7 $$\begin{aligned} \sum _t x_{it} = 1, \quad \quad \forall \ i \in \mathcal {A}. \end{aligned}$$

We relax these constraints for all activities and turn them into one penalty *P*1 denoted by the expression (8). To ensure that the search for an optimal solution penalizes any infeasible solution, the expression is squared so that satisfying the constraint gives no penalty to the QUBO (i.e., remains 0), and that violating the constraint increases the objective value (which we try to minimize).

8 $$\begin{aligned} P1(x) = \sum _{i=0}^{n+1} \left( \sum _t x_{it} - 1 \right) ^2 . \end{aligned}$$

We can model the precedence constraints of two consecutive activities using their inequalities (9).

9 $$\begin{aligned} \sum _{t \in \mathcal {H}} t x_{jt} \le \sum _{t \in \mathcal {H}} t x_{it} + p_i \quad \quad \forall \ (i,j) \in \mathcal {E}. \end{aligned}$$

The reformulation of all precedence constraints into a single penalty, denoted as $P2$, results in (10). Here, the need to square the decision variables does not arise since any quadratic expression involving two binary variables never yields a negative value. It is easy to understand that the only scenario where such a constraint is not satisfied occurs when two consecutive activities erroneously start at the same time.

10 $$\begin{aligned} P2(x) = \sum _{(i,j) \in \mathcal {E}} \sum _{t \in \mathcal {H} } \sum _{\begin{array}{c} t' \in \mathcal {H} \\ t' \backslash t + p_i > t' \end{array}} x_{it} x_{jt'} . \end{aligned}$$

The JSSP has machine-sharing constraints which can also be modeled by simple quadratic expressions, thus giving a penalty similar to *P*2. The RCPSP has another difficulty: resource constraints. The inequalities presented in (11) express these constraints, as modeled in^51^.

11 $$\begin{aligned} \sum _{i=1}^n b_{ik} \sum _{\tau = t - p_i + 1}^t x_{i \tau } \le B_k \quad \forall t \in \mathcal {H}, \forall k \in R . \end{aligned}$$

Since we need a penalty for relaxing the resource constraints, we add slack variables in order to reach an equality for each inequality of (11). Slack variables are noted $z_{tk}, t \in \mathcal {H}, k \in R$, as a non-negative integer for reformulating resource constraints (11) with the quadratic penalties (12).

12 $$\begin{aligned} P3(x) = \sum _t \sum _k \left( \sum _{i=1}^n b_{ik} \sum _{\tau = t - p_i + 1}^t x_{i \tau } - B_k + z_{tk} \right) ^2. \end{aligned}$$

Since this reformulation aims to create a QUBO, the slack variables $s_{tk}$ must correspond to binary variables. Here, we can consider the minimum value of $z_{tk}$ as zero and the maximum value as $B_k$. The related binary expression is given by equation (13), where *y* is a binary vector (each $y^i$ takes value in {0,1}) and where the integer function $f(\alpha )$ gives the required maximum power of 2 with $\alpha$ as the target integer.

13 $$\begin{aligned} z_{tk} = \sum _{i=0}^{f(B_k)} 2^i y^i \quad \forall t \in \mathcal {H}, \forall k \in \mathcal {R}. \end{aligned}$$

We note that $\lambda _1$, $\lambda _2$, and $\lambda _3$ multipliers balance the penalties *P*1, *P*2, *P*3, respectively. The QUBO of the RCPSP can be formulated using the objective function $f_{\text {QUBO}}(x)$ with the quadratic constraints (8), (10), and (12), such that $f_{\text {QUBO}}(x) = f(x) + \lambda _1 P1(x) + \lambda _2 P2(x) +\lambda _3 P3(x)$, as presented in equation (14).

14 $$\begin{aligned} \begin{aligned} f_{\text {QUBO}}(x)&= \sum _t t x_{(n+1)t} \\&\quad + \lambda _1 \sum _{i=0}^{n+1} (1 - \sum _t x_{it})^2 \\&\quad + \lambda _2 \sum _{(i,j) \in \mathcal {E}} \sum _t \sum _{t' \backslash t + p_i > t'} x_{it} x_{jt'} \\&\quad + \lambda _3 \sum _t \sum _k (\sum _{i=1}^n b_{ik} \sum _{\tau = t - p_i + 1}^t x_{i \tau } - B_k + z_{tk})^2 . \end{aligned} \end{aligned}$$

### Experimental results

**Figure 11:**
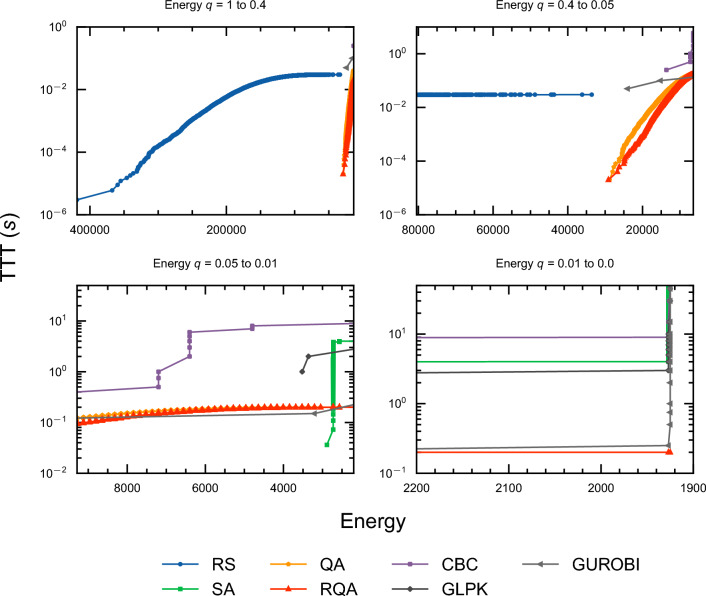
Evolution of TTT in seconds for a cumulative instance (OS = 0.1) of six non-dummy activities and two resources. The three panels show the evolution for different energy ranges, where energy decreases from left to right. The right-most panel shows the energy range for the ground state of this instance. From the same panel, it can be seen that RQA is able to find the ground state energy equivalent to 1925 with a TTT of 0.2 seconds.

**Figure 12:**
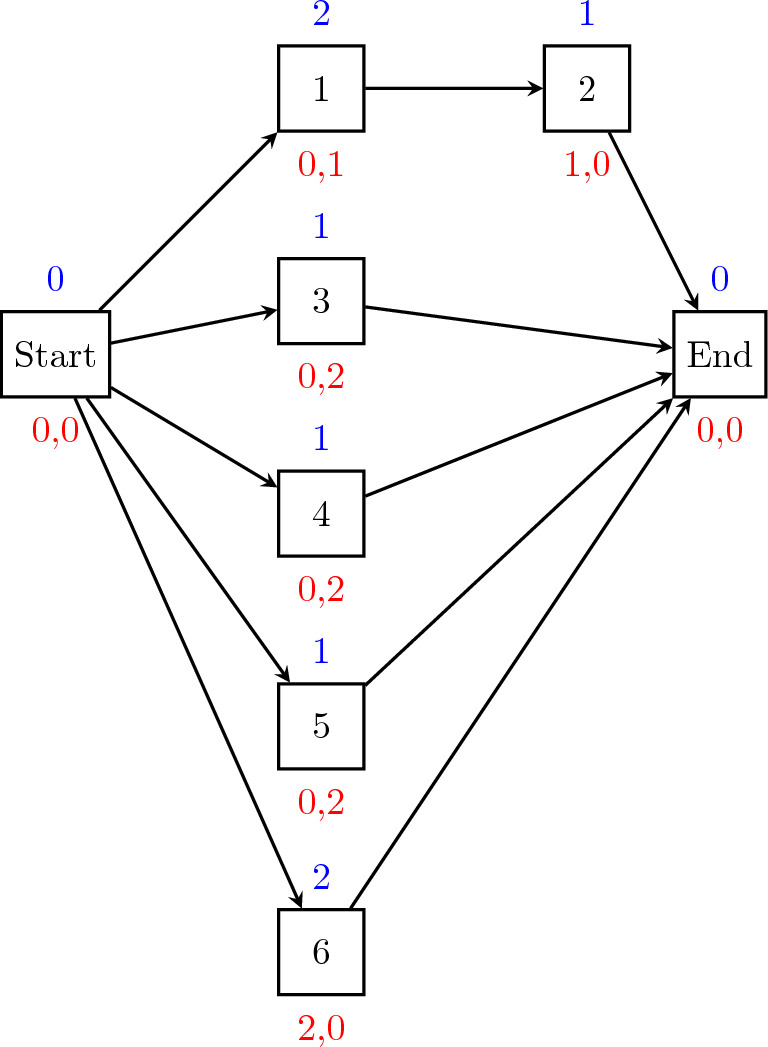
Network diagram cumulative instance (OS = 0.1) with six non-dummy activities and two resources. The duration of each activity is displayed above each node, while the resource consumption is displayed below.

Figure 11 illustrates the TTT evolution for a range of optimization methods applied to a specific cumulative instance involving six non-dummy activities and two resources. Accompanying this, Fig. 12 presents the network diagram relevant to this instance. The figures are organized into a tripartite panel, demonstrating the progression of the optimization process across different energy quantiles. This progression is depicted from left to right, indicating a transition from higher to lower energy levels.

It is noteworthy that QA and RQA exhibit discernible patterns, contrasting with the Random Sampling (RS) behavior observed in the evolution curve of RS. This observation suggests that QA and RQA are not mere random samplers.

**Figure 13:**
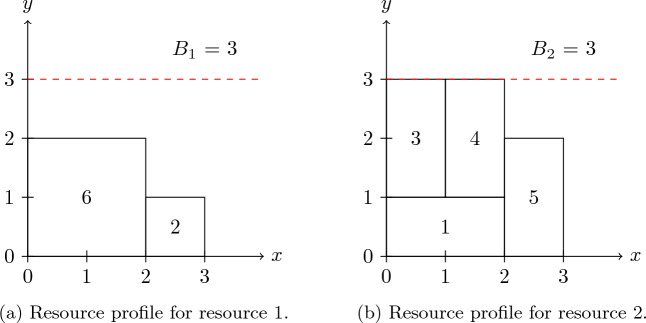
Optimal schedule obtained from RQA for the cumulative instance (OS = 0.1) with six non-dummy activities and two resources, described in Fig. 11. This optimal solution was obtained in a TTT of 0.2 seconds. The optimal schedule corresponds to a ground state energy of 1925. Please see table I4 in the appendix I for detailed values of the ground state energies for each instance.

For the specific instance presented in Fig. 11, RQA demonstrates exceptional performance and outperforms all other optimization methods, including the widely used commercial solver GUROBI. This superior performance of RQA is particularly noticeable in the third panel of Fig. 11, where, the distinctive red line marked with “$\triangle$” symbols, representing RQA, reaches the ground state energy faster than any other method. Furthermore, Fig. 13 illustrates the optimal schedule derived using RQA. This schedule results in an optimal project make span of three days.

**Figure 14:**
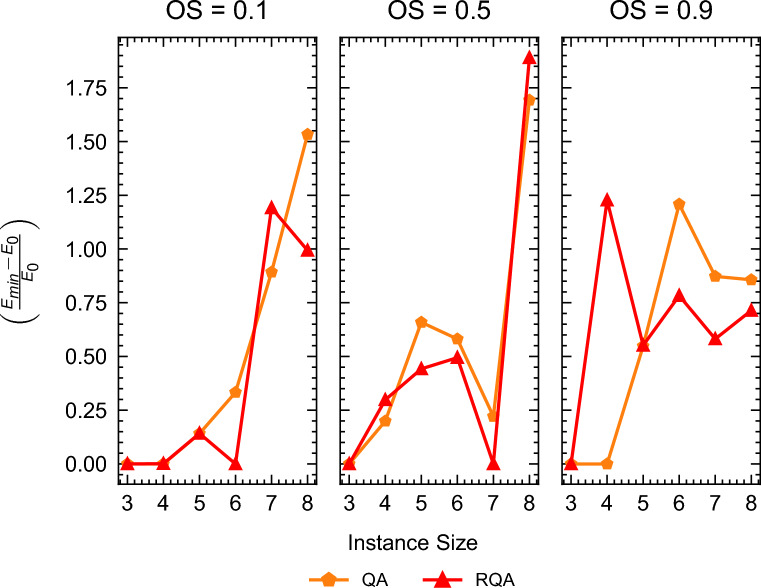
Relative energy deviation from the ground state measured as $\left( \frac{E_{\min } - E_0}{E_0}\right)$ for all instance types (Cumulative, Medium OS, and Disjunctive) for both QA and RQA. A value of $\left( \frac{E_{\min } - E_0}{E_0}\right) = 0$ indicates that the ground state energy was achieved. Ground state energy values of the different instance types and sizes can be seen in table I4 from the Appendix I.

Despite the impressive performance of QA and RQA for the instance depicted in Fig. 11, it is crucial to remember that both QA and RQA are heuristic methods and consequently do not guarantee the finding of ground states or global optimal solutions. This limitation is evident in Fig. 14, which showcases the relative deviation $\left( \frac{E_{\min } - E_0}{E_0}\right)$ between the minimum energy solution obtained by QA and RQA (i.e., $E_{\min }$) and the ground state energy (i.e., $E_0$) obtained by Gurobi. A value of $\left( \frac{E_{\min } - E_0}{E_0}\right) = 0$ in this metric indicates a successful sampling of the ground state. However, as illustrated in Fig. 7, both QA and RQA struggled to find the ground state energy solutions. Additional data in Figures H11 and H12 in Appendix H demonstrate the time-to-solution (TTS) in seconds for QA and RQA respectively.

**Figure 15:**
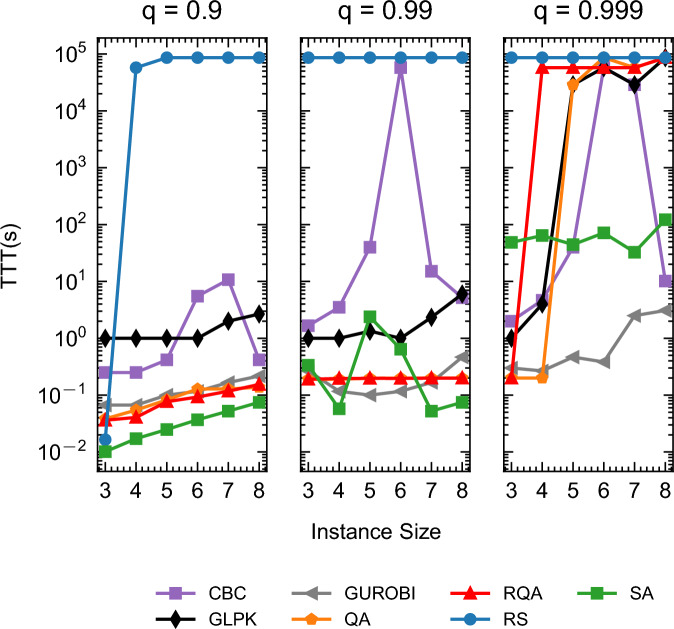
Mean TTT, reported in seconds for all instance types; OS = 0.1, OS = 0.5, and OS = 0.9.

Table 3 presents succinct results featuring the mean TTT values for solutions at different energy quantiles (0.9, 0.99, and 0.999). To provide further insight, Fig. 15 visually captures the distinctive mean TTT patterns and highlights the performance variations among the evaluated optimization methods for all instance types. Figure D2, D3, and D4 from Appendix D offer further insight by illustrating the individual TTT performance for the different instance types evaluated in this work (cumulative, medium OS, and disjunctive).

**Table 3:** TTT (s) mean results for disjunctive, cumulative, and medium OS instances.

Size	Method	$q=0.9$	$q=0.95$	$q=0.99$	$q=0.999$	$q=0.9999$
3	CBC	0.25	0.25	1.67	2	2
3	GLPK	1	1	1	1	1
3	GUROBI	0.07	0.07	0.27	0.3	0.3
3	QA	0.04	0.09	0.19	0.2	0.2
3	RQA	0.04	0.08	0.19	0.2	0.2
3	RS	0.02	57600	86400	86400	86400
3	SA	0.01	0.01	0.34	48.60	59.66
4	CBC	0.25	0.25	3.5	4.67	4.67
4	GLPK	1	1	1	4	28801.33
4	GUROBI	0.07	0.07	0.12	0.27	0.5
4	QA	0.05	0.12	0.19	0.20	28800.13
4	RQA	0.04	0.11	0.20	57600.07	86400
4	RS	57600.01	86400	86400	86400	86400
4	SA	0.02	0.02	0.06	64.20	93.92
5	CBC	0.42	0.67	40	40	45
5	GLPK	1	1	1.33	28802	57601
5	GUROBI	0.1	0.1	0.1	0.47	0.75
5	QA	0.08	0.16	0.20	28800.13	86400
5	RQA	0.08	0.16	0.20	57600.07	86400
5	RS	86400	86400	86400	86400	86400
5	SA	0.02	0.02	2.39	44.41	136.76
6	CBC	5.5	28803.17	57603	57603	57615
6	GLPK	1	1	1	57601	57610
6	GUROBI	0.12	0.12	0.12	0.38	2.17
6	QA	0.13	0.17	0.20	86400	86400
6	RQA	0.09	0.15	0.20	57600.07	86400
6	RS	86400	86400	86400	86400	86400
6	SA	0.04	0.04	0.64	71.46	159.07
7	CBC	10.75	15.08	15.08	28810.08	28810.08
7	GLPK	2	2	2.33	28820	86400
7	GUROBI	0.17	0.17	0.17	2.5	16.17
7	QA	0.13	0.18	0.20	57600.07	86400
7	RQA	0.12	0.17	0.20	57600.07	57600.07
7	RS	86400	86400	86400	86400	86400
7	SA	0.05	0.05	0.05	32.65	108.17
8	CBC	0.42	0.42	5.17	10.17	28800.17
8	GLPK	2.67	2.67	6	86400	86400
8	GUROBI	0.22	0.22	0.47	3.08	5
8	QA	0.14	0.18	0.20	86400	86400
8	RQA	0.15	0.18	0.20	86400	86400
8	RS	86400	86400	86400	86400	86400
8	SA	0.07	0.07	0.07	121.81	441.25

The results presented in Table 3 and Fig. D4, D2, and D3 reveal interesting dynamics. It is evident that QA and RQA face challenges in maintaining superiority, especially when tasked with finding ground states in instances of larger sizes. Despite this, in mere fractions of a second, both QA and, notably, RQA demonstrate their capability to provide high-quality solutions approaching the proximity of ground states.

Overall, for the high-energy quantiles, GUROBI exhibits consistent superiority over SA, QA, RQA, and other free solvers. However, this trend does not extend to certain freely available solvers, such as GLPK and CBC. Notably, in scenarios involving lower energy quantiles, both QA and RQA demonstrate significant advantages over these free solvers. This is particularly evident in the left ($q=0.9$) and middle ($q=0.99$) panels of Fig. 15, where QA and RQA outperform their counterparts in reaching lower energy states more efficiently.

Contrary to the findings reported by Carugno et al.^12^ in the context of the JSSP, our results indicate that SA surpasses both QA and RQA in terms of performance across most instance sizes and energy quantiles. In agreement with their observations, however, RQA does tend to show superior performance compared to QA, particularly in lower energy quantiles.

**Figure 16:**
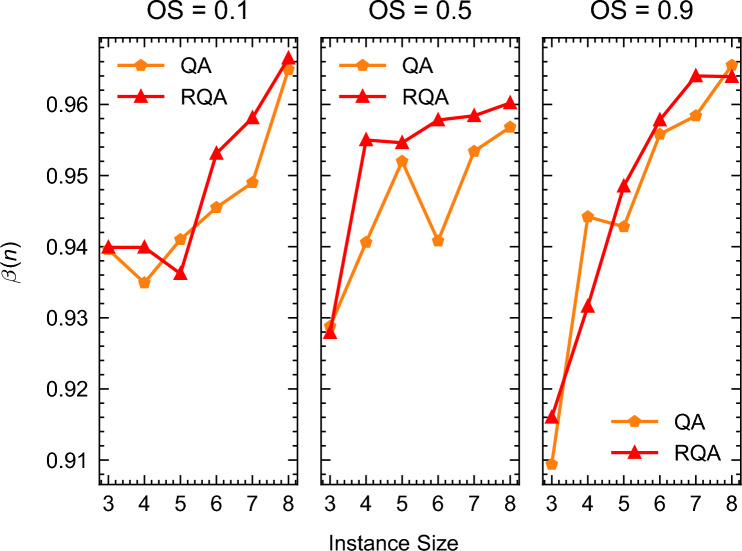
$\beta (n)$ for all instances with 3, 4, 5, 6, 7, and 8 non-dummy activities for all instance types (Cumulative, Medium OS, and Disjunctive).

Adapting the Q-score methodology for QA, as proposed by Van der Schoot et al.^67^, we computed the $\beta (n)$ values for various instance sizes and types examined in our study. Analysis of Fig. 16 reveals that the $\beta (n)$ values for both QA and RQA consistently exceed the critical threshold of 0.2. However, is important to remind the reader that this threshold was empirically established based on the solution to instances of the the Max-Cut problem using QAOA. Therefore, its validity is based solely on these empirical findings and is without additional theoretical support.

We, therefore, conclude that the estimated maximum instance size of the RCPSP that can be solved using a D-Wave quantum annealer peaks at seven non-dummy activities. Although the $\beta (n)$ values substantially exceed the critical threshold, we observe a rapid decline in solution quality beyond this activity count. This deterioration is evident when examining the mean “chain-break” percentage reported by the D-Wave system. For instances with eight activities or more, the average chain-break percentage escalates to approximately 74%, indicating a significant breakdown among the logical qubits that were utilized to map the original QUBO graph onto the QPU topology. With such a high percentage of chain breaks, the reliability of the annealer in solving the intended optimization problem becomes questionable, despite the higher $\beta (n)$ values observed.

### Anneal time and pausing effects

In the preceding section, our experimental approach was predicated on determining an optimal annealing time for QA. This determination was made empirically, analyzing the performance of QA across a spectrum of annealing times: 1, 5, 10, 20, 50, and 100$\mu s$.

The performance evaluation of QA for each annealing time was determined based on a relative energy difference metric. This metric juxtaposes the minimum energy achieved through QA against the ground state energy for the given instances as $\left( \frac{E_{\min } - E_0}{E_0}\right)$. Our analysis spanned instance with 3, 4, 5, 6, 7, and 8 non-dummy activities and all instance types (Cumulative, Medium OS, and Disjunctive). We collected 1000 samples for each configuration.

**Figure 17:**
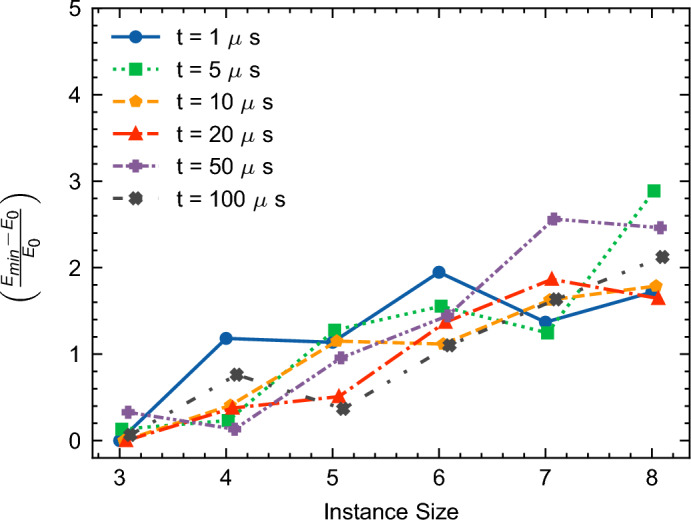
Mean effect of annealing time on QA performance, measured as the mean relative deviation from the ground state $\left( \frac{E_{\min } - E_0}{E_0}\right)$ for all instance types (Cumulative, Medium OS, and Disjunctive). A value of 0 on the y-axis indicates an energy equal to the ground state energy. Please see table I4 in the appendix I for detailed values of the ground state energies for each instance.

Insights into the mean behavior of QA across these different annealing times are presented in Fig. 17. This line graph illustrates the relationship between the instance size (x-axis) and the relative energy differences (y-axis), offering a visual representation of the performance variations across instance sizes.

**Figure 18:**
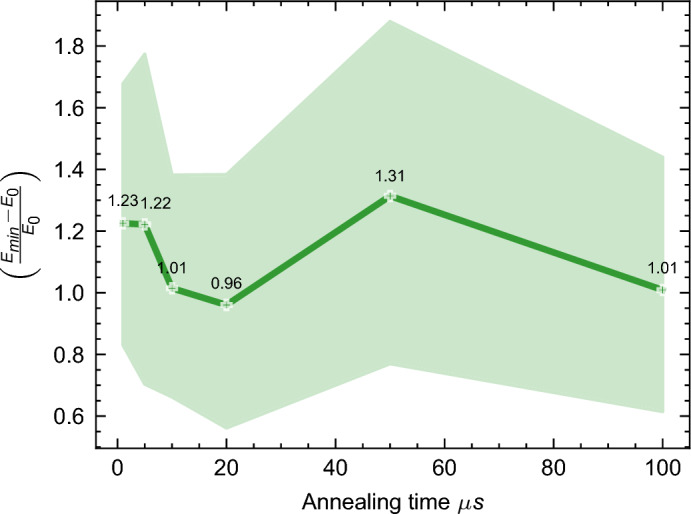
The figure illustrates the impact of annealing times on QA performance, depicted across various instance sizes and types as a function of the mean relative deviation from the ground state $\left( \frac{E_{\min } - E_0}{E_0} \right)$ on the y-axis. This relationship is represented by a green line, accompanied by its standard deviation delineated as a light green contour. The x-axis quantifies the annealing time. A value of 0 on the y-axis indicates an energy equal to the ground state energy. Please see table I4 in the appendix I for detailed values of the ground state energies for each instance.

Complementing this analysis, Fig. 18 presents a holistic view of the mean behavior across all instance sizes and types. Here, the annealing times are mapped along the x-axis, and the corresponding relative energy differences are charted on the y-axis. In accordance with^71^, this graph reveals that an annealing time of approximately 20$\mu s$ emerges as optimal. Beyond this threshold, there is a discernible decline in QA performance.

Given these findings, we chose to conduct our experiments with an annealing time fixed at 20$\mu s$. This decision was guided by empirical evidence, suggesting that this duration strikes an effective balance in optimizing QA performance. Figures E5, E6, and E7 illustrate the effect of annealing times for Cumulative, Medium OS, and Disjunctive instance types.

In continuation of our exploration into the multiple parameters involved in QA, we extended the investigation to assess the impact of annealing pauses on QA performance. This analysis aligns with the relative energy difference framework established earlier, comparing the minimum energy achieved via QA to the ground state energy for a particular instance.

Our focus centered on a fixed annealing time of 20$\mu s$ to examine the effects of pauses at intervals of 2, 4, 8, 12, 16, and 18$\mu s$ (refer to Fig. 8 for a visual representation of the annealing schedules after incorporating these pauses).

**Figure 19:**
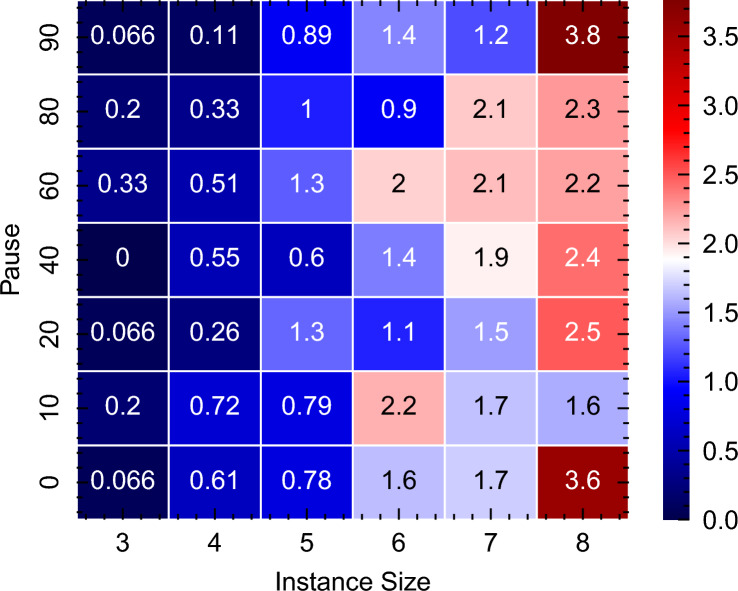
Heatmap of the effect of pauses included in the annealing schedule vs the relative mean deviation from the ground state energy $\left( \frac{E_{\min } - E_0}{E_0}\right)$ for all instance types. The y-axis shows the percentage of pause in the annealing schedule, as shown in Fig. 8. A value of 0, represented by dark blue in the heatmap, indicates an energy equal to the ground state. Please see table I4 in the appendix I for detailed values of the ground state energies for each instance.

This investigation was inspired by the findings of Marshal et al.^77^, who report performance enhancements in QA with the inclusion of annealing pauses. Our results, illustrated in Fig. 19, confirm this observation. Figure 19 presents a heat map that encapsulates the QA performance across various instance sizes (3, 4, 5, 6, 7, and 8 non-dummy activities) and pause durations (shown on the y-axis as the percentage of pause relative to the total annealing time). Each cell in this heat map represents the average relative energy value derived from 1000 samples across all instance types (Cumulative, Medium OS, and Disjunctive), providing a comprehensive view of the performance landscape.

**Figure 20:**
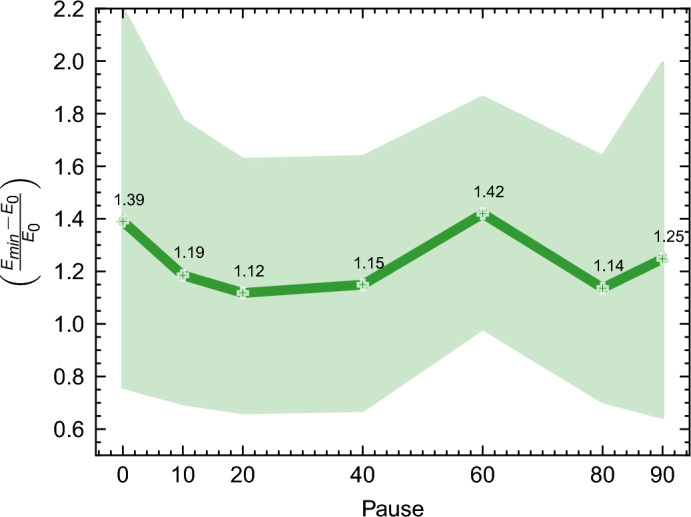
General effect of pauses included in the annealing schedule vs the relative deviation from the ground state energy $\left( \frac{E_{\min } - E_0}{E_0}\right)$ for all instances. The y-axis shows the percentage of pause in the annealing schedule, as shown in Fig. 8.

Intriguingly, Fig. 20 highlights that annealing pauses constituting 20% to 40% of the overall schedule (equating to 4$\mu s$ and 8$\mu s$ in this context) yield the most favorable performance. Figure F8, F9, and F10 show the effect of annealing pauses for Cumulative, Medium OS, and Disjunctive instance types, respectively.

It is important to note, however, that the inherent nature of RQA always incorporates a pause in the annealing schedule. Consequently, the insights obtained from this analysis predominantly apply to conventional QA rather than RQA.

## Discussion

In this work, we have conducted a comprehensive exploration into the practical implementation of the RCPSP using quantum annealing, specifically by leveraging D-Wave’s quantum-computing technology. Our investigation encompasses a range of aspects, from the basics of quantum annealing to advanced techniques like reverse quantum annealing.

To the best of our knowledge, we are the first to address the RCPSP using a quantum annealer, marking a significant contribution to the integration of quantum computing in the field of operations research. Our work underscores the importance of QUBO modeling for solving RCPSP instances on quantum annealing machines. We have conducted a thorough analysis of 12 well-known MILP formulations for RCPSP and converted them to a QUBO format. This includes identifying the most suitable formulation for quantum annealing, specifically the PRI69 formulation, and providing the corresponding QUBO model.

It is worth noting that the QUBO model (14) derived in “RCPSP QUBO” section can easily be adapted to other variants of the RCPSP, as in the case of the 1-preemptive resource-constrained project scheduling problem (1_PRCPSP). By adapting the formulation proposed by Ballestín et al.^78^, we obtain the QUBO $f(x)^{\text {1\_PRCPSP}}_{\text {QUBO}}$ (presented in Appendix G1). The problem does not suffer from the addition of new slack variables. The new constraints can be modeled as a quadratic product that must remain zero in order to be satisfied.

Other versions of the RCPSP can be based on the QUBO introduced in this study without the need to add many constraints. For example, if resources have periods of unavailability, or if their capacity is reduced ($B_k$ becomes $B_{kt}$), it is sufficient to treat these periods as distinct activities with fixed variable values, as shown by Hartmann^79^. This approach can also be applied when the use of resources needs to be stopped without a pre-determined period (e.g., for maintenance operations). In such cases, these periods of unavailability should be considered as activities without setting fixed variables. In these scenarios, it is important to establish a precedence constraint for each virtual activity to ensure that the identification of the unavailability period is properly included in the optimization process. However, this modification will require the inclusion of additional slack variables associated with the resource constraint.

In this work, we solve three distinct categories of problem instances, as defined by the protocol of Baptiste and Le Pape, using RCPSP QUBO formulation (14) on a quantum annealer. Strategies such as developing a reverse quantum annealing schedule are employed. The results are then compared with those obtained from a classical solver. For this comparison, we conduct a detailed exploration of multiple metrics available for evaluating the performance of quantum annealing. Ultimately, we adopt the TTT and the Q-score for our evaluation. Additionally, we determine the maximum instance size that can still be solved with a machine equipped with 5000 qubits. Another key discovery from our research underscores the potential benefits of quantum annealing, especially in scenarios with constrained time limits, such as those encountered in online operations research problems. In addition to our primary research focus, we conduct an extensive and meticulous analysis to understand the impact of annealing times and the role of pauses within annealing schedules on the performance of QA.

While our research offers valuable insights, it’s important to recognize its limitations. These include using problem instances of limited size, while excluding other promising quantum-computing techniques like QAOA^73^ due to hardware constraints, as well as the dependence on the “minor-miner” heuristic for the embedding procedure.

In alignment with the work of Venturelli et al.^11^ and Carugno et al^12^ on the JSSP, we recognize the potential for significant reduction in time horizons for the RCPSP. This reduction can be achieved by pre-establishing upper bounds, derived from heuristics that can be calculated in polynomial time^80^. Such bounds can considerably reduce the number of variables required by time-index formulations like “PRI69”. However, in this study, our focus was centered on examining the intrinsic performance of QA and RQA without incorporating these heuristic techniques. We also did not explore the use of broken-chain correction techniques like the ones suggested by Marshal et al.^32^.

These aspects warrant consideration in future research. Additionally, investigating alternative quantum-computing technologies, such as the neutral atoms technology proposed by Pasqal^81^, is promising and deserves further exploration.

Despite the acknowledged limitations, this work serves as a pioneering effort in applying quantum annealing to the RCPSP. We hope that our findings and methodologies will act as a catalyst for future benchmarks, fostering advancements towards practical applications of quantum computers in operations research.

### Supplementary Information

Supplementary Information.

## Data Availability

The input data and instances to replicate our results are made available in the following online directory: https://github.com/ceche1212/QA_RCPSP.

## Code availability

The code to replicate our results are made available in the following online directory: https://github.com/ceche1212/QA_RCPSP.
