# Supplementary material for: Solving the resource constrained project scheduling problem with quantum annealing
Source: Sci Rep. 2024 Jul 22;14:16784. doi: 10.1038/s41598-024-67168-6 (PMC11263701; doi:10.1038/s41598-024-67168-6)
Supplement: Supplementary file 1 — Supplementary Information. [file 41598_2024_67168_MOESM1_ESM.pdf]

Solving the Resource Constrained Project Scheduling Problem with Quantum Annealing

Luis Fernando PÉREZ ARMAS[1*†], Stefan CREEMERS[1,3†] and Samuel DELEPLANQUE[2†]

[1*]Operations Management, IESEG School of Management, Univ. Lille, CNRS, UMR 9221 - LEM - Lille Economie Management, 3 rue de la digue, Lille, F-59800, Nord, France.
[2]Univ. Lille, CNRS, Centrale Lille, Junia, Univ. Polytechnique Hauts-de-France, UMR 8520 - IEMN, 41 Bd Vauban, Lille, 59000, France.
[3]ORSTAT KU Leuven, Naamsestraat 69, Leuven, 3000, Belgium.

*Corresponding author(s). E-mail(s): l.perezarmas@ieseg.fr;
Contributing authors: s.creemers@ieseg.fr; stefan.creemers@kuleuven.be; samuel.deleplanque@junia.com;
†These authors contributed equally to this work.

Supplementary material

Appendix A UML activity diagram: resolution process with a quantum annealing solver

The Unified Modeling Language (UML) activity diagram presented in Fig. A1 summarizes the process of solving an optimization problem, such as the RCPSP, by implementing QA on a quantum machine. The activities are divided into two sets: on the left, human activities corresponding to problem modeling, and on the right, those automatically executed by the machine. The machine takes as input either an Ising model or a QUBO model. Therefore, if the user only has a MILP, as is the case in this work for 12 different formulations, they must formulate it either as an Ising model or a QUBO model. If the latter is provided, the machine automatically converts it to Ising, as the two models are isomorphic. In addition, the user also provides weights corresponding to the penalties of each series of relaxed constraints (i.e., the multipliers λ_i for each constraint i).

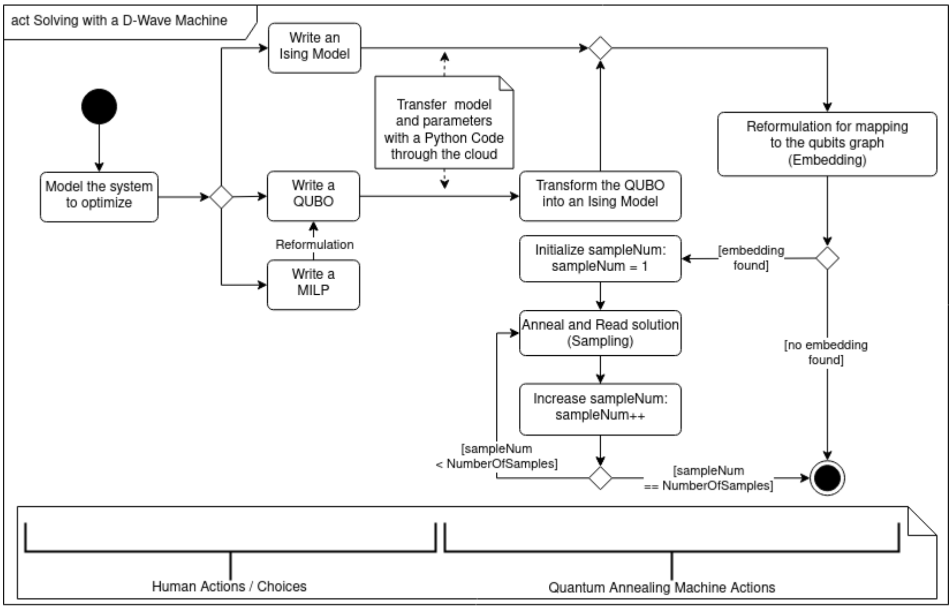

Fig. A1: UML activity diagram: transitioning from the mathematical model to the quantum annealing solution process. Each activity belongs either to the actions to be performed by the user or to the actions to be executed by the machine. The user provides a QUBO or an Ising model to the machine, and it replicates the QA as many times as requested by the user, resulting in an equivalent number of solutions obtained at the end of the process.

Once the machine has an Ising model to process, the corresponding non-directed graph must be adapted to the machine's topology, that is, the qubits network. Thus, the Ising model is transformed into another non-directed graph suited to the topology. The resulting graph is generally less dense in order to match the machine's connectivity. It uses more qubits for this purpose ("embedding" process). At the end of this process, if the machine does not find an "embedding", the experiment stops. Otherwise, the QA process is applied to the mapped graph.

The number of QA replications is determined by the number of *anneals* and *reads*, collectively referred to as *samples*. Users must specify two key parameters for each QA experiment conducted on a D-Wave machine: the number of *samples* and the time available for each anneal. These parameters are crucial and significantly influence the outcome of the experiment. Given that each experiment is typically restricted to a duration of about one second, the total time available for quantum machine access is primarily allocated between these two parameters. The optimal balance between them is usually determined empirically and varies depending on the specific optimization problem being addressed.

In terms of output, the user obtains as many solutions as the executed anneals. Even if all obtained solutions are feasible, in the sense that the variable type is respected (binary) and no constraint violates the QUBO and Ising models, the feasibility of the solutions must be verified through the constraints of the initial MILP model, an operation that can be done in polynomial time.

Appendix B List of Symbols

Symbol	Definition
$\mathcal{H}(t)$	Time-dependent energy Hamiltonian
$\mathcal{H}_0$	Tunneling Hamiltonian
$\mathcal{H}_1$	Problem Hamiltonian
$A(t)$	Annealing schedule function associated with $\mathcal{H}_0$
$B(t)$	Annealing schedule function associated with $\mathcal{H}_1$
$\mathcal{P}$	Optimization Problem
G	Network graph of $\mathcal{P}$
V	Nodes of G
E	Edges of G
s_j	Spin variable $\in \{-1, 1\}$, associated with qubit j
h_j	Linear term of the Ising model, associated with spin variable s_j
$J_{i,j}$	Interaction term of the Ising model, associated with spin variables s_i and s_j
x_j	QUBO Binary variable $\in \{0, 1\}$ associated with qubit j
n	Number of project activities
$\mathcal{A}$	Set of project activities
$\mathcal{E}$	Set of precedence constraints
p_j	Duration of project activity j
$b_{j,k}$	Resource consumption of activity j for resource k
$\mathcal{R}$	Set of project renewable resources
$\mathcal{S}$	Project solution schedule
S_j	Start date of activity j in Schedule $\mathcal{S}$
B_k	Maximum capacity of resource k
$\mathcal{H}$	Set of available time-slots t
$\mathcal{T}$	Planning horizon (max value of t
$\beta(n)$	Q-Score ratio
β^*	Q-score critical ratio threshold
$\tilde{C}(n)$	Average energy value obtained from solutions of QA for problem $\mathcal{P}$
$C_{max}(n)$	minimum solution value of problem $\mathcal{P}$
$C_r(n)$	Average energy value obtained from solutions of RS for problem $\mathcal{P}$
n^*	Max number of qubits for which $\beta(n) \geq \beta^*$
Q	QUBO Matrix
$Q_{i,j}$	QUBO Interaction coefficient of variables i and j in matrix Q
$\mathcal{X}$	set of binary QUBO variables
x	QUBO solution
$E(x)$	Total QUBO energy of solution x
$v(x)$	QUBO objective function energy of solution x
$c(x)$	QUBO energy contribution of constraints for solution x
λ	QUBO Penalty term
W_c	Verma and Lewis penalty function
s	Standardized forward annealing schedule progress. 0 is no progress and 1 is 100% progress.
E_{min}	Minimum energy obtained from solution method when solving problem $\mathcal{P}$
E_0	Ground state energy of problem $\mathcal{P}$
$\mathcal{F}$	Forbidden sets of `TAM93`
$\mathcal{I}$	Feasible sets of `MIN98`
$\phi^k_{i,j}$	`ART03` Resource flow between activities i and j for resource k
t	time (integer units)
t_e	scheduled start-time for event e (Formulations of `KON11` and `ART13`)
$z_{t,k}$	Slack variables used for resource constraints of RCPSP QUBO
ρ	Chain strength parameter

Table B1: List of Symbols

Appendix C Evaluation of MILP formulations for RCPSP

Table C2: Evaluation of MILP formulations for RCPSP.

Instance	Type	Family	MILP	n MILP	n (QUBO)	S (QUBO)	ρ
10_H	Disjunctive	Time Index	PRI69	570	1136	566	0.1566
10_H	Disjunctive	Sequence	TAM93	110	2321	2161	0.0073
10_H	Disjunctive	Sequence	ART03	310	3316	2356	0.0094
10_H	Disjunctive	Event	ART13	230	5559	5219	0.0056
10_H	Disjunctive	Event	KON11	111	4824	4658	0.0070
10_L	Cumulative	Time Index	PRI69	380	830	450	0.1740
10_L	Cumulative	Sequence	TAM93	110	2328	2168	0.0073
10_L	Cumulative	Sequence	ART03	310	3329	2369	0.0093
10_L	Cumulative	Event	ART13	230	4724	4384	0.0068
10_L	Cumulative	Event	KON11	111	4235	4069	0.0080
15_H	Disjunctive	Time Index	PRI69	990	1758	768	0.1443
15_H	Disjunctive	Sequence	TAM93	240	7681	7351	0.0022
15_H	Disjunctive	Sequence	ART03	690	9859	7729	0.0035
15_H	Disjunctive	Event	ART13	495	17678	17003	0.0021
15_H	Disjunctive	Event	KON11	241	15360	15023	0.0026
15_L	Cumulative	Time Index	PRI69	1035	1851	816	0.1526
15_L	Cumulative	Sequence	TAM93	240	7699	7369	0.0022
15_L	Cumulative	Sequence	ART03	690	9869	7739	0.0035
15_L	Cumulative	Event	ART13	495	16553	15878	0.0023
15_L	Cumulative	Event	KON11	241	15036	14699	0.0027
20_H	Disjunctive	Time Index	PRI69	1780	2840	1060	0.1318
20_H	Disjunctive	Sequence	TAM93	420	17645	17105	0.0009
20_H	Disjunctive	Sequence	ART03	1220	21435	17695	0.0017
20_H	Disjunctive	Event	ART13	860	39033	37933	0.0010
20_H	Disjunctive	Event	KON11	421	35028	34481	0.0012
20_L	Cumulative	Time Index	PRI69	1740	2868	1128	0.1427
20_L	Cumulative	Sequence	TAM93	420	17715	17175	0.0009
20_L	Cumulative	Sequence	ART03	1220	21443	17703	0.0017
20_L	Cumulative	Event	ART13	860	36653	35553	0.0011
20_L	Cumulative	Event	KON11	421	34426	33879	0.0012

n MILP = number of variables original problem, n (QUBO) = number of qubits, S (QUBO) = number of slack qubits, ρ = density of the QUBO

Table C3: Evaluation of MILP formulations for RCPSP.

Instance	Type	MILP	n MILP	n (QUBO)	S (QUBO)	ρ
10_H	Disjunctive	PRI69	570	1136	566	0.157
10_H	Disjunctive	CHR87	570	3432	2862	0.041
10_H	Disjunctive	SOU97	570	2316	1746	0.009
10_H	Disjunctive	KLE98	570	2803	2290	0.009
10_H	Disjunctive	KLE00_1	570	3673	3103	0.010
10_H	Disjunctive	KLE00_2	1140	3447	2307	0.006
10_H	Disjunctive	BIA13	1710	8016	2886	0.016
10_H	Disjunctive	MIN98	684	1187	503	0.099
10_L	Cumulative	PRI69	380	830	450	0.174
10_L	Cumulative	CHR87	380	2932	2552	0.034
10_L	Cumulative	SOU97	380	1542	1162	0.010
10_L	Cumulative	KLE98	380	1354	1012	0.023
10_L	Cumulative	KLE00_1	380	2735	2355	0.014
10_L	Cumulative	KLE00_2	760	2288	1528	0.013
10_L	Cumulative	BIA13	1140	5342	1922	0.023
10_L	Cumulative	MIN98	646	1611	965	0.065
15_H	Disjunctive	PRI69	990	1758	768	0.1443
15_H	Disjunctive	CHR87	990	7694	6704	0.022
15_H	Disjunctive	SOU97	990	4065	3075	0.006
15_H	Disjunctive	KLE98	990	6726	5802	0.009
15_H	Disjunctive	KLE00_1	990	6199	5209	0.008
15_H	Disjunctive	KLE00_2	1980	6027	4047	0.004
15_H	Disjunctive	BIA13	2970	13965	5055	0.013
15_H	Disjunctive	MIN98	1320	2796	1476	0.046
15_L	Cumulative	PRI69	1035	1851	816	0.153
15_L	Cumulative	CHR87	1035	8913	7878	0.020
15_L	Cumulative	SOU97	1035	4389	3354	0.006
15_L	Cumulative	KLE98	1035	5029	4063	0.011
15_L	Cumulative	KLE00_1	1035	9372	8337	0.007
15_L	Cumulative	KLE00_2	2070	6441	4371	0.008
15_L	Cumulative	BIA13	3105	14739	5424	0.012
15_L	Cumulative	MIN98	2001	5054	3053	0.055
20_H	Disjunctive	PRI69	1780	2840	1060	0.132
20_H	Disjunctive	CHR87	1780	15945	14165	0.014
20_H	Disjunctive	SOU97	1780	7177	5397	0.004
20_H	Disjunctive	KLE98	1780	15366	13675	0.005
20_H	Disjunctive	KLE00_1	1780	13492	11712	0.005
20_H	Disjunctive	KLE00_2	3560	10709	7149	0.003
20_H	Disjunctive	BIA13	5340	24977	8957	0.010
20_H	Disjunctive	MIN98	2581	5570	2989	0.030
20_L	Cumulative	PRI69	1740	2868	1128	0.143
20_L	Cumulative	CHR87	1740	18997	17257	0.012
20_L	Cumulative	SOU97	1740	7624	5884	0.004
20_L	Cumulative	KLE98	1740	11391	9738	0.007
20_L	Cumulative	KLE00_1	1740	19217	17477	0.004
20_L	Cumulative	KLE00_2	3480	11076	7596	0.006
20_L	Cumulative	BIA13	5220	25024	9364	0.009
20_L	Cumulative	MIN98	5307	12620	7313	0.089

n MILP = number of variables original problem, n (QUBO) = number of qubits, S (QUBO) = number of slack qubits, ρ = density of the QUBO

Appendix D TTT supplementary plots

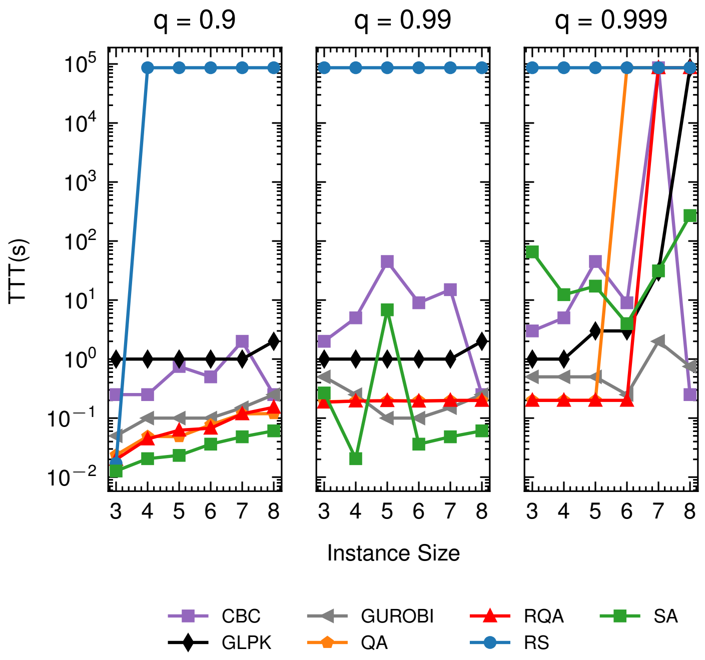

Fig. D2: Time-to-Target, reported in seconds for Cumulative instances with OS = 0.1.

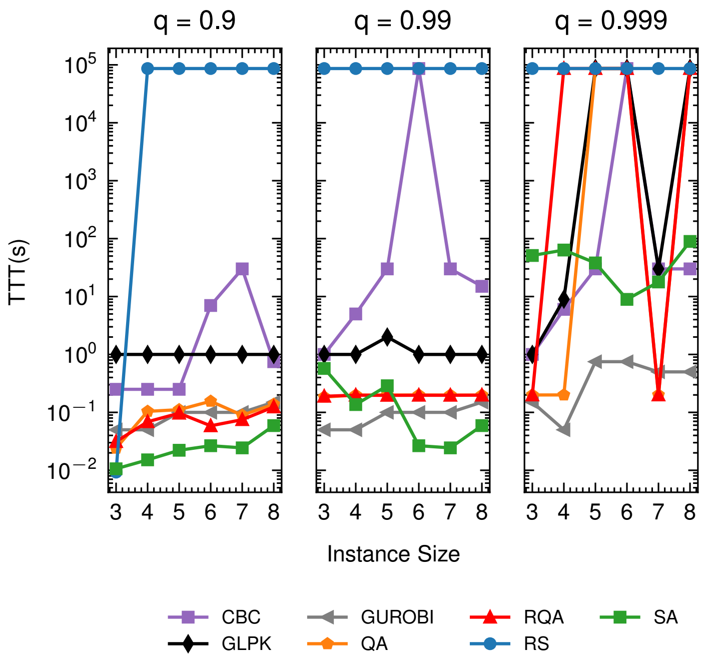

Fig. D3: Time-to-Target, reported in seconds for Medium OS instances with OS = 0.5.

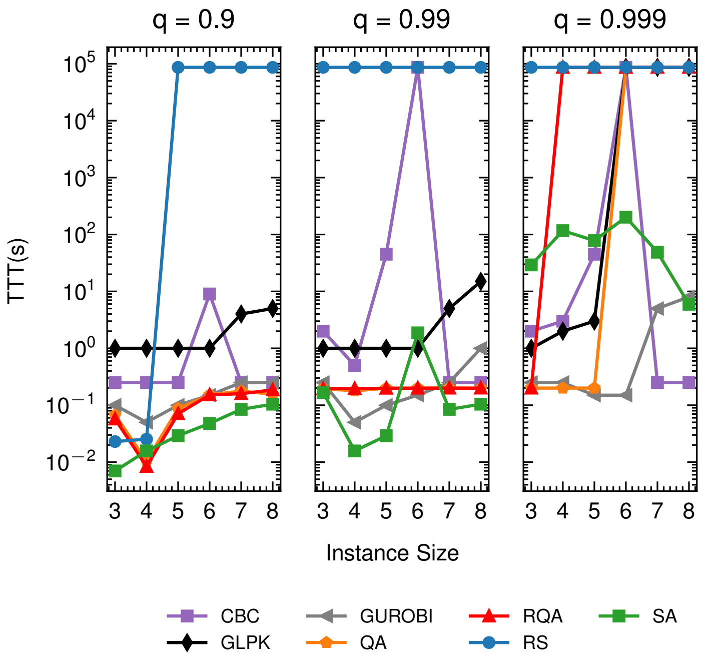

Fig. D4: Time-to-Target, reported in seconds for Disjunctive instances with OS = 0.9.

Appendix E Annealing time supplementary plots

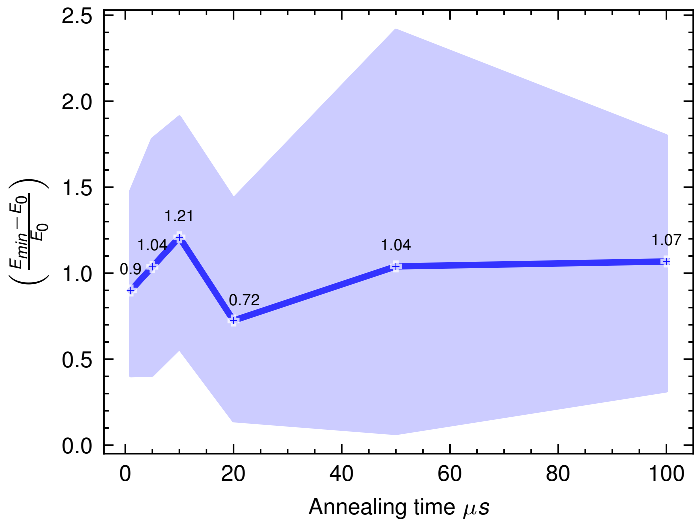

Fig. E5: Overall effect of annealing time on QA performance, measured as the mean relative deviation from the ground state $\left(\frac{E_{\min}-E_0}{E_0}\right)$ for Cumulative instances and all sizes (i.e., 3, 4, 5, 6, 7, and 8 non-dummy activities).

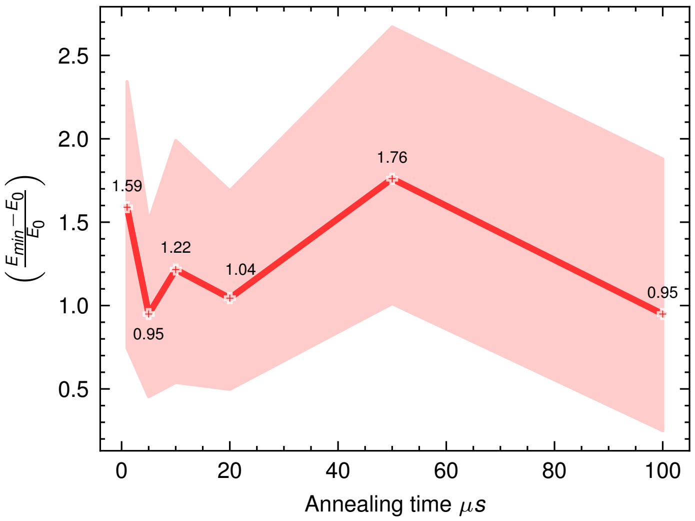

Fig. E6: Overall effect of annealing time on QA performance, measured as the mean relative deviation from the ground state $\left(\frac{E_{\min}-E_0}{E_0}\right)$ for Medium OS instances with various sizes (i.e., 3, 4, 5, 6, 7, and 8 non-dummy activities).

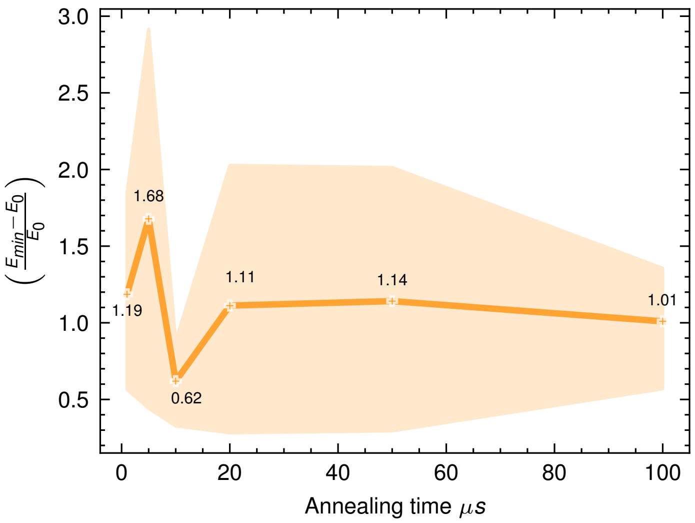

Fig. E7: Overall effect of annealing time on QA performance, measured as the mean relative deviation from the ground state $\left(\frac{E_{\min}-E_0}{E_0}\right)$ for Disjunctive instances and all sizes (i.e., 3, 4, 5, 6, 7, and 8 non-dummy activities).

Appendix F Annealing pauses supplementary plots

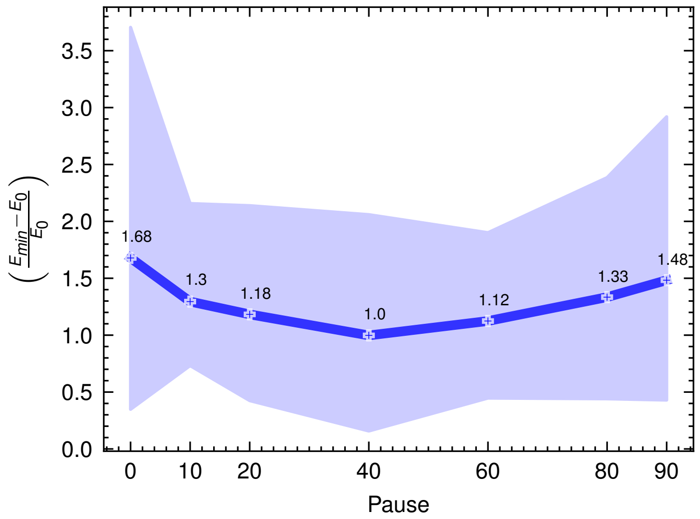

Fig. F8: General effect of pauses included in the annealing schedule vs the relative deviation from the ground state energy $\left(\frac{E_{\min}-E_0}{E_0}\right)$ for Cumulative instances. The y-axis shows the percentage of pause in the annealing schedule.

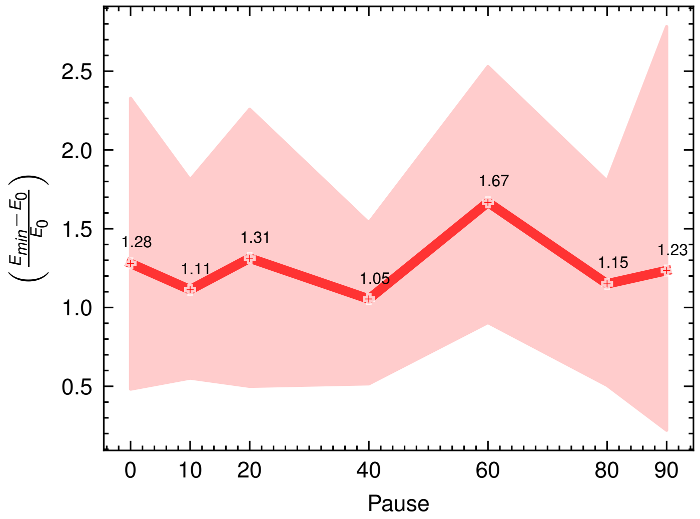

Fig. F9: General effect of pauses included in the annealing schedule vs the relative deviation from the ground state energy $\left(\frac{E_{\min}-E_0}{E_0}\right)$ for Medium OS instances. The y-axis shows the percentage of pause in the annealing schedule.

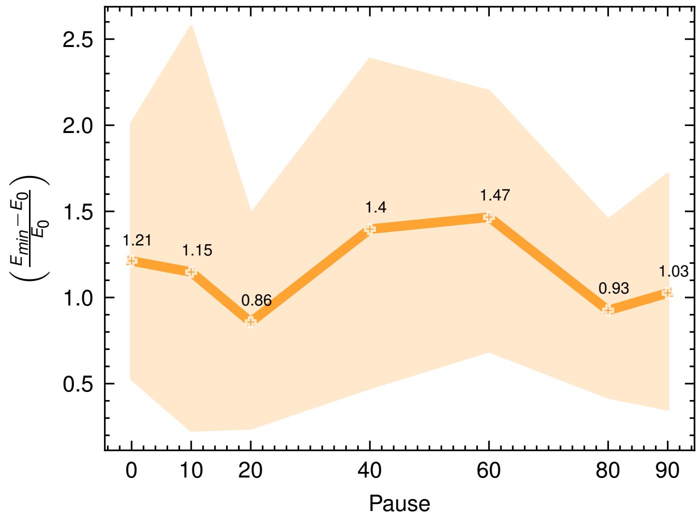

Fig. F10: General effect of pauses included in the annealing schedule vs the relative deviation from the ground state energy $\left(\frac{E_{\min}-E_0}{E_0}\right)$ for Disjunctive instances. The y-axis shows the percentage of pause in the annealing schedule.

Appendix G QUBO of the 1_PRCPSP

$$\begin{aligned} f(x)^{\text{1\_PRCPSP}}_{\text{QUBO}} &= \sum_t t x_{(n+1)t} \\ &+ \lambda_1^A \sum_{i=0}^{n+1} \left(1 - \sum_t x_{iAt}\right)^2 \\ &+ \lambda_1^B \sum_{i=0}^{n+1} \left(1 - \sum_t x_{iBt}\right)^2 \\ &+ \lambda_2^{BB} \sum_{(i,j)\in\mathcal{E}} \sum_t \sum_{t' \setminus t+p_{iB}>t'} x_{iBt} x_{jBt'} \\ &+ \lambda_2^{AB} \sum_{i\in\mathcal{A}} \sum_t \sum_{t' \setminus t+p_{iA}>t'} x_{iAt} x_{iBt'} \\ &+ \lambda_3^C \sum_t \sum_k \left(\sum_{i=1}^n b_{ik} \sum_{\tau=t-p_{iA}+1}^{t} x_{iA\tau} + x_{iB\tau} - B_k + s_{tk} \right)^2. \end{aligned} \tag{G1}$$

We adapt the notation of the multipliers according to the activity and the type of constraints (e.g. "λ_1^B"). Each original variable x_{it} is now divided into two parts x_{iAt} and x_{iBt} that must comply with:

$$\sum_{t\in\mathcal{H}} t x_{iAt} \leq \sum_{t\in\mathcal{H}} t x_{iBt} + p_A \qquad \forall i \in \mathcal{A}.$$

Appendix H TTS Supplementary plots

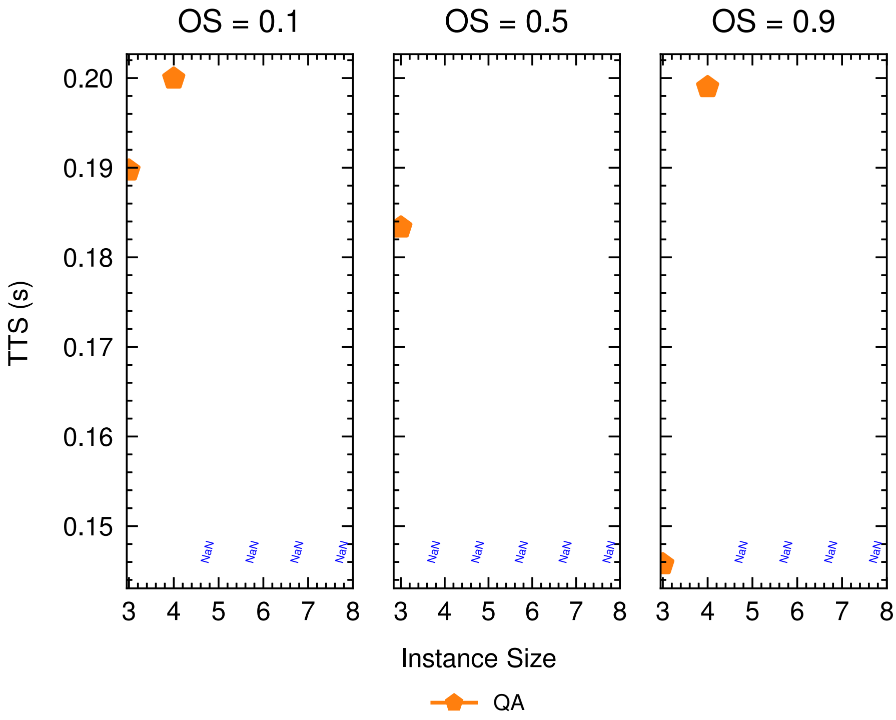

Fig. H11: Quantum Annealing (QA) Time-to-Solution, reported in seconds for all instance types; OS = 0.1, OS = 0.5, and OS = 0.9.

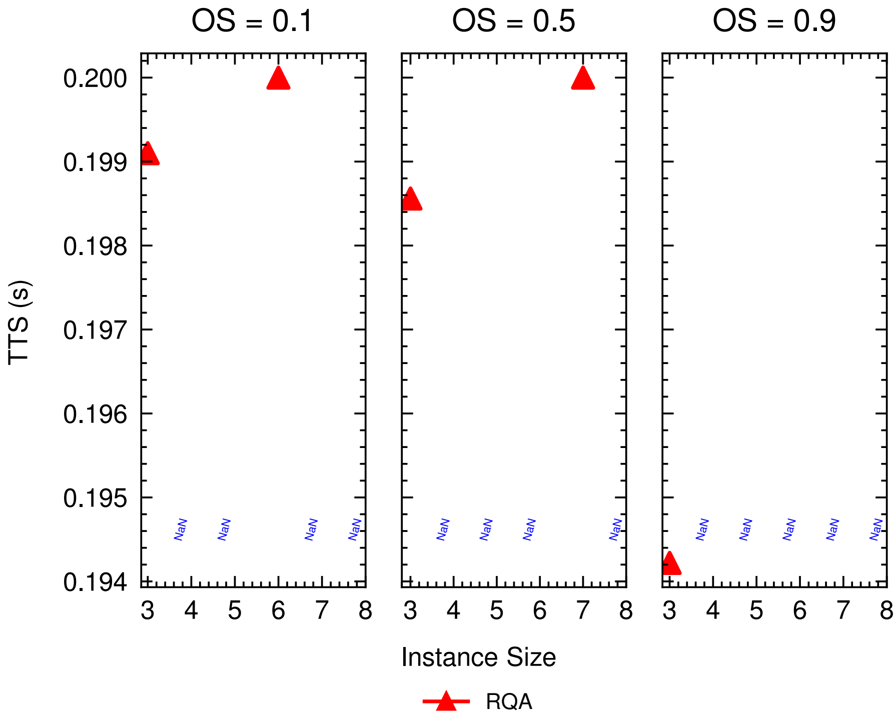

Fig. H12: Reverse Quantum Annealing (RQA) Time-to-Solution, reported in seconds for all instance types; OS = 0.1, OS = 0.5, and OS = 0.9.

Appendix I Ground state energies

Table I4: Ground state energies for all instance types and sizes

Size	Type	E_0
3	Cumulative	605
3	Disjunctive	483
3	Middle	304
4	Cumulative	845
4	Disjunctive	485
4	Middle	1607
5	Cumulative	986
5	Disjunctive	1447
5	Middle	1628
6	Cumulative	1925
6	Disjunctive	3090
6	Middle	2168
7	Cumulative	2008
7	Disjunctive	6733
7	Middle	1447
8	Cumulative	2870
8	Disjunctive	6722
8	Middle	2009

Ground state energy E_0 for each instance size and for all instance types; OS = 0.1 (Cumulative), OS = 0.5 (Middle), and OS = 0.9 (Disjunctive).
